# Vaccination against COVID-19 reduces virus-related fears: Findings from a German longitudinal study

**DOI:** 10.3389/fpubh.2022.878787

**Published:** 2022-07-28

**Authors:** Daniel Seddig, Dina Maskileyson, Eldad Davidov

**Affiliations:** ^1^Faculty of Management, Economics and Social Sciences, Institute of Sociology and Social Psychology, University of Cologne, Cologne, Germany; ^2^Faculty of Law, Institute of Criminal Law and Criminology, University of Münster, Münster, Germany; ^3^University Research Priority Program “Social Networks”, University of Zurich, Zürich, Switzerland

**Keywords:** COVID-19, vaccination, fear of COVID-19, mental health, differences-in-differences

## Abstract

During the recent pandemic, fear of COVID-19 has been widespread and is considered to deteriorate mental health. We assessed whether vaccination can effectively reduce the fear of COVID-19 and, thus, contribute to improving people's mental health status. We used two-wave panel data from a German online study collected in April 2021 (t1) and August/September 2021 (t2) and estimated differences-in-differences to determine whether those who were vaccinated against COVID-19 experienced a reduction of fear of COVID-19, and whether the reduction was more evident as compared to people who were not vaccinated for various reasons. Fear of COVID-19 generally decreased between t1 and t2 for all respondents. Moreover, reduction of fear for people who were unvaccinated at t1 but received the vaccine between t1 and t2 was strongest as compared to people who did not get vaccinated during that period, even after we controlled for factors associated with fear (e.g., age, gender). Vaccination reduced fear of COVID-19 beyond some seasonal fluctuation and, therefore, we argue that vaccination partially reduces the psychological distress caused by the pandemic. We recommend that scientists, practitioners, and politicians highlight this additional positive effect of vaccination in health communication.

## Introduction

The COVID-19 pandemic has sparked concerns around the globe. People express these concerns as fear of negative health consequences, hospitalization, and dying from an infection (1, 2). Moreover, fear of COVID-19 increases general levels of psychological distress, post-traumatic stress symptoms, panic disorder, insomnia, anxiety, and depression (1–4). At the same time, fear of COVID-19 propels compliance with regulations to contain the virus and increases the willingness to get vaccinated (5–7). Does vaccination lead to a sense of security? If yes, a reduction in the fear of COVID-19 should be measureable when comparing levels of fear of individuals who seized the opportunity and received a vaccination once it was available with the levels of fear of individuals who did not. Since 2021, the possibility to receive an injection of any of the approved vaccine types was granted on a priority basis according to age, health issues, contact with vulnerable groups, and working in system-relevant professions (8). By late summer 2021, every resident wishing to be vaccinated had received the opportunity to do so. Yet, according to the data from the Robert Koch Institute, by November 2021 only about 70% of the eligible population had used this opportunity. Thus, strategies to further disseminate the vaccines and increase vaccination willingness are needed.

Social and personal reactions to the pandemic and related fears and concerns are strongly linked to information processes, and it is therefore useful to coordinate the efforts to improve vaccination programs and developments of effective reactions to the pandemic (9). Especially in the early stages of the pandemic, such efforts had to circumvent misinformation (and even disinformation) before developing useful reactions. By now, the evidence regarding the effect of the virus and vaccination on peoples' fear of COVID-19 is more compelling. For example, recent studies have indicated that fear of COVID-19 is related to various mental health outcomes such as anxiety and depression and even higher suicide risk (3, 4, 10). A recent systematic review and meta-analysis has reported excessive levels of fear of COVID-19 around the world (2). Another comprehensive review demonstrated that different population groups tend to experience different levels of fear (1). Specifically, women, younger adults, urban residents, divorced people, healthcare workers, as well as people in quarantine settings, people with suspected COVID-19 infection, and people with mental health problems were found to be at risk of excessive fear of COVID-19. These findings suggest that higher fear of COVID-19 should increase vaccination intentions and encourage the population to follow national vaccination recommendations. However, some studies also report a reversed effect, that is, vaccination decreases fear of COVID-19 (11–13). Accordingly, people who received the COVID-19 vaccine were more likely to report decreased mental distress levels afterwards than their unvaccinated counterparts. Thus, receiving the vaccine results in improvements in mental health.

The current study aims at exploring whether and to what extent people in Germany vaccinated against COVID-19 decreased their level of fear of the virus as compared to their unvaccinated counterparts. We use panel data (i.e., data from the same individuals) across two time points (late April 2021 and late August/early September 2021), which allows us to conduct a (quasi-experimental) “pre-post testing” analysis. In addition, we distinguish the following groups of individuals according to their vaccination status: (1) individuals vaccinated between the first and the second measurement time point; (2) individuals vaccinated prior to the first time point; (3) vaccination refusers, namely, individuals unvaccinated by the second measurement time point, reporting that they “do not want to be vaccinated”; and (4) unvaccinated individuals at the second measurement time point for other reasons such as “not received an offer” or “not yet arranged an appointment”. We expect differences in levels of fear of COVID-19 between these groups. The highest level of fear of COVID-19 is expected for people who got vaccinated either between the first and second measurement time points (group 1) and for people vaccinated prior to the first measurement time point (group 2). We assume that people with a higher initial level of fear were more likely to take the opportunity to do something to alleviate their fears and get vaccinated (*Hypothesis 1*). By way of contrast, and for the same reason, we expect the level of fear of COVID-19 of vaccination refusers (group 3) to be lowest (*Hypothesis 2*). Finally, we expect people in group 1—after receiving the first vaccination—experienced the highest reduction of fear of COVID-19 as compared to other groups (*Hypothesis 3*).

## Materials and methods

### Study design and participants

We used an ongoing German online access panel by the market research institute Respondi AG (14) to collect the data repeatedly from the same participants. Thus, the design of our study is a prospective panel design, and the data reflect current changes in the characteristics under study. However, participants also provided some information retrospectively, such as their vaccination status since the last data collection. Study participants were recruited online via campaigns, marketers, and by self-recruitment and, after registering, participants received an e-mail invitation to take part in the study voluntary. They did not sign a separate consent form for this study, and they received an incentive of 0.75 euro for their participation. The company used quotas for gender, age, education, income, and immigration background to achieve comparable rates in the sample to those in the German population (15). The share of people with an immigration background in the sample (17%) was below the share provided by German microcensus data (25%). All other quotas were met. The study was approved by the ethics committee of the Faculty of Management, Economics and Social Sciences, University of Cologne (reference numbers: 210005DS and 210031DS).

The first wave (t1) of data was collected between April 9 and April 28, 2021 and the second wave (t2) was collected between August 23 and September 9, 2021, addressing the same participants. Of the 5,044 respondents who participated in the first wave, 3,458 were re-interviewed in the second wave. In this study, we focused only on the respondents who participated in both waves and provided valid information on their vaccination status and fear of COVID-19. Thus, the effective size of the two-wave panel sample was 3,428. In this sample, 27% of the respondents were aged 60 or above (mean age 49), 54% were male, 23% reported a polytechnic or University degree of education, 29% reported having low income (a maximum of 2,000 € per month), and 17% reported having an immigrant background (with at least one parent born outside of Germany). The rates of people aged 60 or above and males were lower in the original first wave sample (23 and 50%, respectively) than in the two-wave panel sample.

Due to the slow start of the vaccination campaign and initial shortages of vaccines, only a small proportion of the German population had received their first vaccination by mid-April 2021 (19.6% on April 17 according to the Robert Koch Institute (RKI) and the Federal Ministry of Health) (16). In our first wave of the data collection, 19.4% of the respondents reported being vaccinated at least once. On June 7, 2021, the German government suspended all vaccination restrictions that prioritized people of older age, with pre-existing health issues, in certain jobs (e.g., health care), and with social and economic disadvantages. Thus, between our first and second data collection, all citizens theoretically had the opportunity to be vaccinated at least once. In our second wave of data collection, 82% of the respondents reported they received at least one vaccination. The official data showed that only 65.7% of the general population were vaccinated once by September 1, 2021 (16). This discrepancy may indicate a selection of vaccinated people into the second wave of the study but it may also reflect that the study sample included only adults aged 18–74 years.

Fear of COVID-19 is the outcome variable of this study. Differences between people that are vaccinated or unvaccinated can be conceptualized as counterfactual states such as

y0 = fear of COVID-19 without vaccination

y1 = fear of COVID-19 with vaccination

meaning that if unvaccinated, fear of COVID-19 is y0 and y1 otherwise. Thus, if the vaccination status does not change across time and everything else being equal across groups, differences in the fear of COVID-19 are solely attributable to group differences and cross-time fluctuations that apply to each group equally. A change in vaccination status is assumed to change the level of fear of COVID-19 over and beyond group differences and cross-time fluctuations. These assumptions correspond to a differences-in-differences (DiD) design for two groups and two time points (17).

Based on the data on vaccination status in our study, we distinguished between four groups across two time points (Table 1): people vaccinated between the first and second waves (group 1); people vaccinated prior to the first wave (group 2); vaccination refusers, namely, unvaccinated until the second wave, reporting that they “do not want to be vaccinated” (group 3); and unvaccinated until the second wave not due to refusal but for other reasons such as “not received an offer” or “not yet arranged an appointment” (group 4). Thus, only group 1 had experienced a change in vaccination status between the waves of data collection. Notably, the group composition was not random, because a substantial proportion of individuals may have self-selected themselves into getting vaccinated as soon as they had the opportunity to receive or refuse a vaccination. In addition, we controlled in our analysis for age, gender, education, income, and immigration status to account for different levels of fear of COVID-19 as a function of these characteristics.

**Table 1:** Study groups and descriptive statistics.

	***N*** **(%)**	**Vaccinated at least once at** *t*_1_**?**	**Vaccinated at least once at** *t*_2_**?**	**Mean** **age**	**%** **Male**	**%** **High education**	**%** **Low income**	**%** **Immi-grants**
Group 1: vaccinated between *t*_1_ and *t*_2_	2,139 (62.40)	No	Yes	49.0	55.4	24.0	27.6	16.6
Group 2: vaccinated prior to *t*_1_	683 (19.92)	Yes	Yes	53.0	55.0	22.5	25.0	13.3
Group 3: refusers	399 (11.64)	No	No	45.4	46.9	15.8	40.1	18.6
Group 4: unvaccinated for other reasons	207 (6.04)	No	No	42.3	51.2	22.2	39.1	24.6
Total sample	3,428 (100.00)			49.0	54.1	22.6	29.3	16.7

*High education, polytechnic or University degree; low income, a maximum of 2,000 €; immigrants, at least one parent born outside of Germany*.

### Measures

We measured fear of COVID-19 using three indicators that resemble a scale that has been developed to assess fear of COVID-19 in the general population (18): (1) “When I think of the coronavirus, I feel threatened” (fear_1_); (2) “I am worried that I or people I love could get sick from the coronavirus” (fear_2_); (3) “I am stressed in the presence of other people, because I am afraid that I may catch the coronavirus” (fear_3_). Respondents were asked to rate these statements on a scale ranging from 1 (strongly disagree) to 7 (strongly agree). All indicators were assessed at both waves (*t*_1_ and *t*_2_), with a negligible missing values rate below 1%. Descriptive statistics for the indicators measuring fear of COVID-19 can be found in the Supplementary Table S1. Subjective measures of fear may require a higher effort of validation and may be less reliable than other measures. However, we had no access to diagnostic (physiological) tests of fear, and we aimed to assess the subjective feeling of an anticipated threat or harm from the virus.

### Statistical analysis

We used confirmatory factor analysis to assess how well the subjective measures capture the construct fear of COVID-19 and to control for measurement error that may compromise the validity and reliability of the results (19). Thus, fear of COVID-19 was treated as a latent variable measured by multiple observed indicators (fear_1_-fear_3_) in multiple groups (group 1-group 4) and across multiple time points (*t*_1_ and *t*_2_). In addition, we tested if the measurements of fear of COVID-19 are invariant across groups and time (20). Measurement invariance is a prerequisite for comparing latent means and latent mean differences across groups and time. It ensures that the group- and time-specific means of a latent variable (i.e., fear of COVID-19) are not biased due to systematic differences in measurement instrument properties across groups and due to systematic shifts in response behavior across time that do not correspond to real differences or change of the construct.

The group- and time-specific latent means were used to calculate the differences-in-differences following the structured means modeling (SMM) approach, (21) which is implemented in structural equation modeling (SEM) (22). Therefore, we included the following differencing equations into the model estimation:

DiD for:      |Equations

group 1 and group 2    |DiD_1_ = (*g_1_t_2_ – g_1_t_1_) – (g_2_t_2_ – g_2_t_1_)*

group 1 and group 3    | DiD_2_ = (*g*_1_*t*_2_ – *g*_1_*t*_1_) – (*g*_3_*t*_2_ – *g*_3_*t*_1_)

group 1 and group 4    | DiD_3_ = (*g*_1_*t*_2_ – *g*_1_*t*_1_) – (*g*_4_*t*_2_ – *g*_4_*t*_1_)

group 2 and group 3    | DiD_4_ = (*g*_2_*t*_2_ – *g*_2_*t*_1_) – (*g*_3_*t*_2_ – *g*_3_*t*_1_)

group 2 and group 4    | DiD_5_ = (*g*_2_*t*_2_ – *g*_2_*t*_1_) – (*g*_4_*t*_2_ – *g*_4_*t*_1_)

group 3 and group 4    | DiD_6_ = (*g*_3_*t*_2_ – *g*_3_*t*_1_) – (*g*_4_*t*_2_ – *g*_4_*t*_1_)

**Figure 1:**
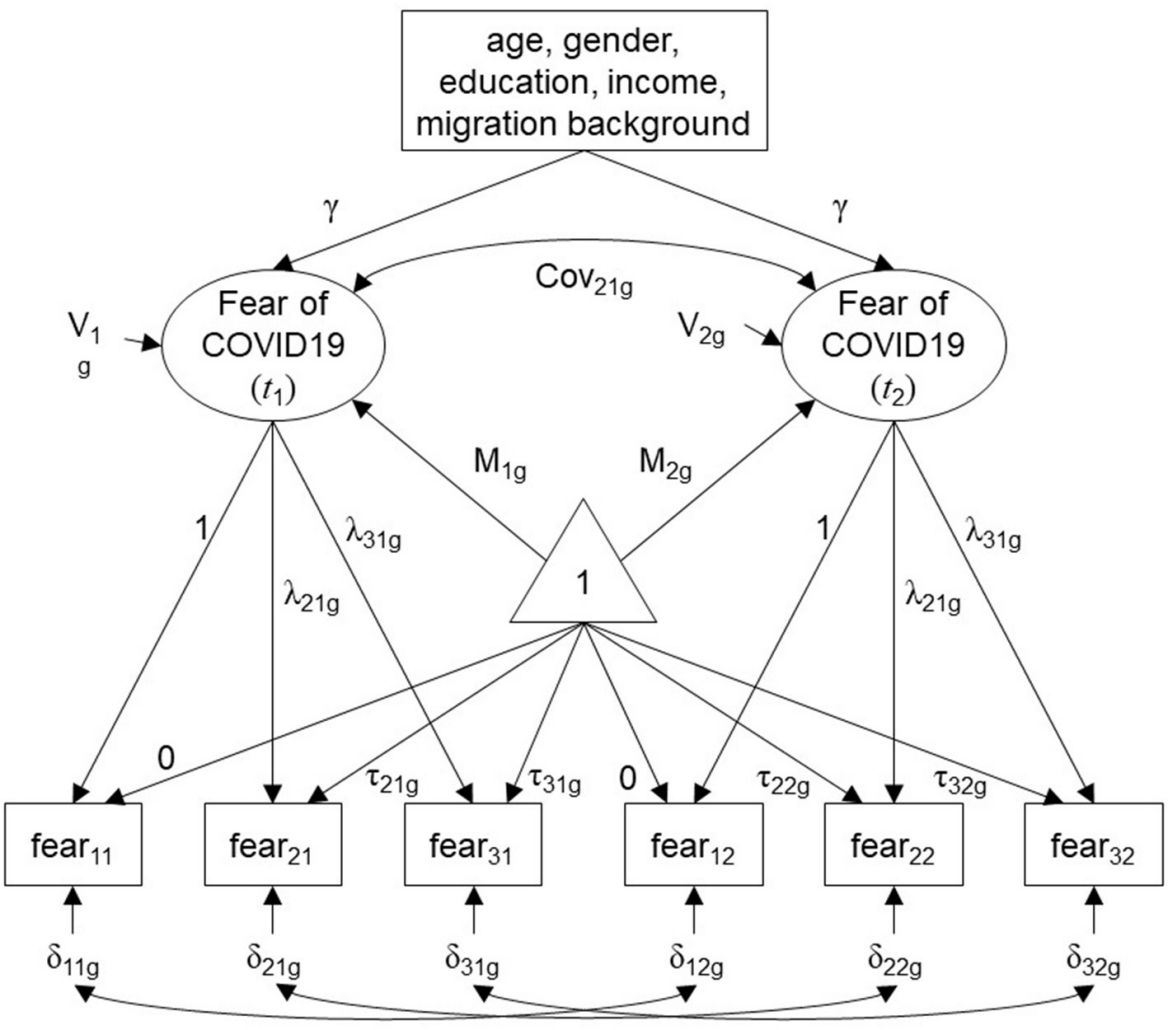
Path diagram displaying the estimated model following the SMM approach. Observed indicators and covariates in rectangles; latent variables in ellipses; subscript “g” refers to the study groups; V, variance of the latent variable; M, mean of the latent variable; Cov, covariance between latent variables; triangle containing “1”, unit-constant pseudo variable capturing the mean structure; γ, coefficients for the regression of fear of COVID-19 on covariates (constrained to be equal across time); λ, factor loading (loadings for indicators fear_11_ and fear_12_ were fixed to 1 for identification); τ, indicator intercept (intercepts for indicators fear_11_ and fear_12_ were fixed to 0 for identification); δ, residual variance.

A path diagram displaying the estimated model is presented in Figure 1.

We assessed the fit of the models to the data by considering standard SEM goodness of fit statistics (23). The chi-square (χ^2^) test statistic tests the null hypothesis that the model-implied and population covariances are equal given the model degrees of freedom (df). However, with larger sample sizes, the power of the χ^2^ test to detect even very small differences between model-implied and population covariances increases, which leads to an excessive rejection of useful models. Therefore, we consider two alternative fit indices based on χ^2^. The comparative fit index (CFI) compares the estimated model to a null model and ranges between 0 and 1. Model fit is considered acceptable when the CFI statistic is close to or above 0.95. The root mean square error of approximation (RMSEA) is a measure of discrepancy of the estimated and a (perfectly fitting) saturated model and ranges between 0 and ∞. Model fit is considered acceptable when the RMSEA statistic is close to or below 0.08.

All models were estimated using the lavaan package in the R environment (24). Estimates were obtained using robust (full-information) maximum likelihood estimation. Annotated R code and output can be found in the Supplementary material.

## Results

First, we estimated an unconstrained confirmatory factor model for fear of COVID-19 at t1 and t2 for the entire sample to assess the factor loadings and reliability (see Supplementary material for details). The model fitted the data well: χ^2^ = 28.432 (df = 5, *p* < 0.001), CFI = 0.998, RMSEA = 0.043. The standardized factor loadings of the indicators measuring fear of COVID-19 were high in magnitude and ranged between 0.740 and 0.911 indicating that a sufficient degree of variance in the observed indicators is explained by the latent variables. Omega reliability coefficients were 0.857 (t1), 0.872 (t2), and 0.893 (total).

Second, we assessed measurement invariance of the latent variable fear of COVID-19. Results indicated that scalar invariance (i.e., equal factor loadings and indicator intercepts) holds between groups and across time simultaneously. This allowed us to make valid comparisons of the latent means of fear of COVID-19 (see Supplementary material for comparisons of fit statistics and further details).

Third, we estimated the model to test the hypothesized latent means and latent mean differences. In this model, again, factor loadings and indicator intercepts were held equal across groups and time. The model showed good fit to the data: χ^2^ = 442.869 (df = 148, *p* < 0.001), CFI = 0.976, RMSEA = 0.049. The standardized factor loadings were between 0.668 and 0.911. Regarding the latent mean level of fear of COVID-19, Table 2 shows that unvaccinated individuals (group 4), those vaccinated between t1 and t2 (group 1), and those vaccinated prior to t1 (group 2) had the highest level of fear of COVID-19 at t1, partially supporting hypothesis 1. Vaccination refusers (group 3) had the lowest level of fear of COVID-19 at t1, supporting hypothesis 2.

**Table 2:** Latent means of fear of COVID-19 across groups and differences between *t*_1_ and *t*_2_.

	**Latent mean** *t*_1_ **(SE)**	**Latent mean** *t*_2_ **(SE)**	**Difference** *t*_2_**–***t*_1_
Group 1: vaccinated between *t*_1_ and *t*_2_	4.83 (0.07)	4.29 (0.07)	−0.54
Group 2: vaccinated before *t*_1_	4.79 (0.11)	4.41 (0.11)	−0.38
Group 3: refusers	2.94 (0.16)	2.61 (0.16)	−0.33
Group 4: unvaccinated for other reasons	4.87 (0.20)	4.55 (0.21)	−0.32

Moreover, fear of COVID-19 decreased across time in all groups. This may be attributed to a seasonal improvement of the situation in Germany between the two waves (25). However, the group-specific decrease was highest for individuals vaccinated between t1 and t2 (group 1) followed by individuals vaccinated prior to t1 (group 2). The decrease in fear for refusers (group 3) and individuals unvaccinated at t2 (group 4) was lowest.

**Figure 2:**
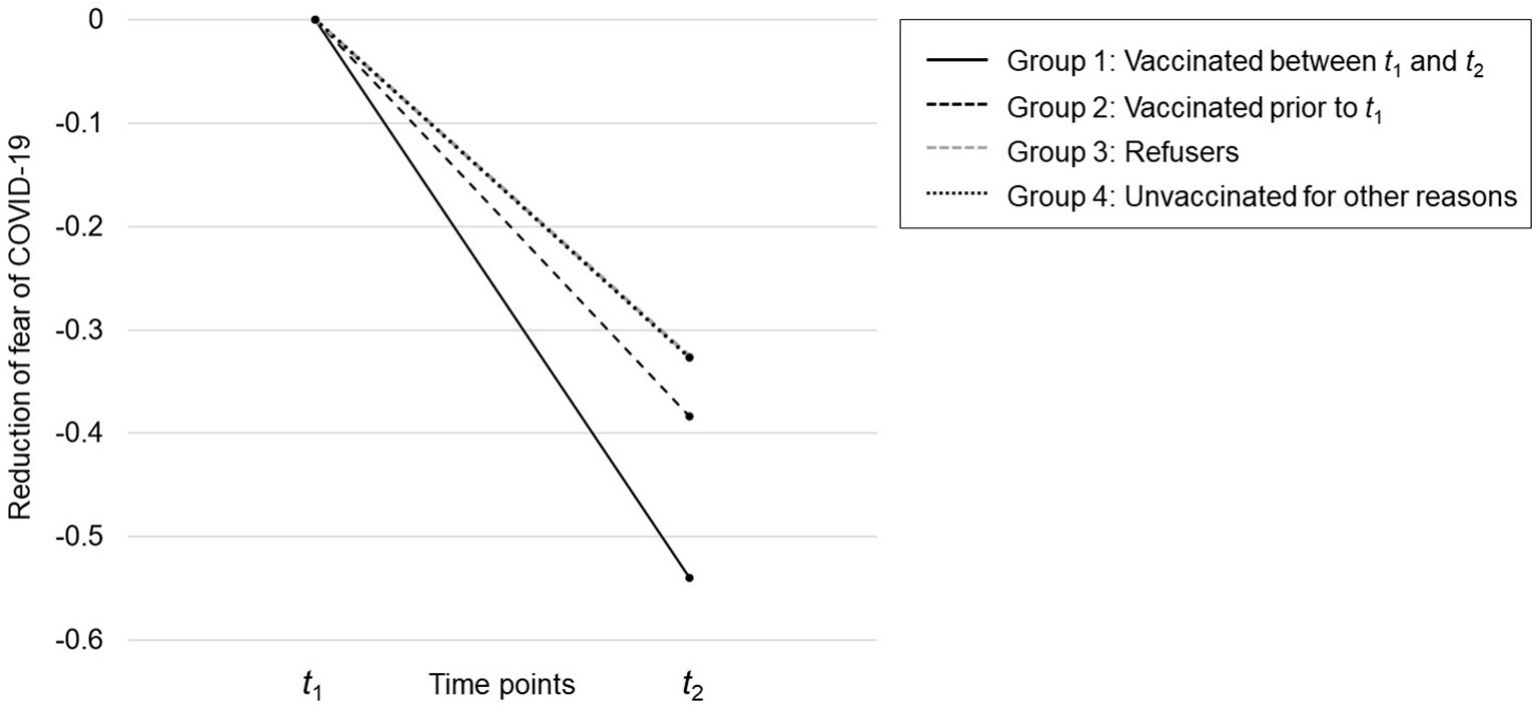
Reduction of fear of COVID-19 across time and between groups. The plotted scores refer to the group-specific differences in the fear of COVID-19 between *t*_1_ and *t*_2_ (see column 4 of Table 2). Thus, each group “starts” at zero. The group-specific differences at *t*_2_ are the differences-in-differences (see Table 3). The scale on the y-axis refers to the scale of the latent variable (fear of COVID-19) at *t*_1_ and *t*_2_, which is similar to the scale of the reference indicator (fear_11_ and fear_12_, respectively).

The differences in the decrease of fear of COVID-19 across groups (i.e., the differences-in-differences) are presented in Table 3. The largest DiD was found between individuals vaccinated between t1 and t2 (group 1) and vaccination refusers (group 3). The DiD between individuals vaccinated between t1 and t2 (group 1) and unvaccinated at t2 for other reasons (group 4) was similar. The DiD between vaccinated individuals between t1 and t2 (group 1) and individuals vaccinated prior to t1 (group 2) was statistically significant. All other differences-in-differences were close to zero. This supports hypothesis 3 and suggests that people who received a vaccination benefitted not only from the vaccination protection but also in terms of their mental health, as their fear of COVID-19 decreased significantly and beyond the general downward trend (see also Figure 2).

**Table 3:** Latent mean differences-in-differences.

	**Latent mean DiD (SE)**	**95% confidence interval**	***p***
Group 1 vs. Group 2 (DiD_1_)	−0.16 (0.06)	(−0.27 −0.05)	0.01
Group 1 vs. Group 3 (DiD_2_)	−0.21 (0.07)	(−0.35 −0.08)	0.00
Group 1 vs. Group 4 (DiD_3_)	−0.21 (0.10)	(−0.41 −0.01)	0.04
Group 2 vs. Group 3 (DiD_4_)	−0.06 (0.08)	(−0.22 0.10)	0.47
Group 2 vs. Group 4 (DiD_5_)	−0.06 (0.11)	(−0.27 0.16)	0.60
Group 3 vs. Group 4 (DiD_6_)	0.00 (0.12)	(−0.23 0.23)	1.00

While assessing the differences in the decrease of fear of COVID-19 across groups we controlled for age, gender, education, income, and immigration status. Some of the control variable effects on fear of COVID-19 were considerable in magnitude but not significant. For the sake of clarity, we only report coefficients with *p* < 0.05 (see the R output for the final model in the Supplementary material for details). People aged 60 or above in group 1 (vaccinated between t1 and t2) experienced higher fear of COVID-19 as did people with low income in group 3 (refusers). The standardized coefficients were 0.16 and 0.22, respectively. Males in group 1 (vaccinated between t1 and t2), group 3 (refusers), and group 4 (unvaccinated for other reasons) experienced less fear of COVID-19. The standardized coefficients were −0.25, −0.23, and 0.29, respectively.

Finally, we tested, in a separate model, if infection status may have influenced fear of COVID-19 at t1 and t2 (see Supplementary material). However, the data on infection status were inconsistent. For example, 29% of those who reported at t1 that they had tested positive, reported at t2 that they had not been tested at all, tested but had no infection, or they did not know. Some respondents may have mistakenly answered this question thinking only about the time since the last interview. Regardless, using a dichotomized infection status dummy (yes/no) for t1 and t2 revealed no meaningful effects on fear of COVID-19 with one exception. People in group 1 (vaccinated between t1 and t2) had higher fear of COVID-19 after testing positive between t1 and t2. This may have driven their decision to get vaccinated.

## Discussion

Since the outbreak of the COVID-19 pandemic, people express their concerns and fears of negative health consequences, hospitalization, and dying from an infection. The fear of COVID-19 is associated with a plethora of negative mental health outcomes, such as psychological distress, post-traumatic stress symptoms, panic disorder, insomnia, anxiety, and depression (1–4). When people experience fear of COVID-19, they are more likely to comply with regulations that are aimed at containing the virus such as getting a vaccination (5–7). In this study we examined whether getting vaccinated in turn leads to a reduction of the fear of COVID-19 that is measureable beyond a general (seasonal) trend. Since the summer of 2021, people in Germany had the opportunity to get vaccinated with different types of vaccines against COVID-19. While many were vaccinated between mid-April and late August/early September 2021, others were not because they refused or did not seek an appointment.

In the current study we explored whether and to what extent those vaccinated against COVID-19 experienced a decrease of fear of COVID-19, and if so, whether the decrease of fear was more evident as compared to others. We assessed study participants' vaccination status, fear of COVID-19, and background variables such as age, gender, education, income, and immigration status at two waves in April (t1) and August/September 2021 (t1). The study allowed the analysis of the data in a (quasi-experimental) “pre-post testing” design and to assess whether the mean level of fear of COVID-19 differed across time and between four groups: group 1—vaccinated between t1 and t2, group 2—vaccinated prior to t1, group 3—refusers, and group 4—unvaccinated for other reasons. Moreover, and most importantly, we tested whether cross-time differences within groups differed between groups by estimating differences-in-differences.

The results partly confirmed our first hypothesis. Many people with a high initial level of fear took the vaccine between t1 and t2 (group 1). However, a small proportion of people with similar initial levels of fear did not get vaccinated for reasons other than refusal (group 4). This group had the lowest mean age and the highest share of people with an immigrant background. Despite their concerns, younger people may not have felt the urgency to get vaccinated in the summer. In addition, access and acceptance barriers may have prevented people with an immigration background to get vaccinated (26).

The lowest level of fear was observed for vaccination refusers (group 3), which confirmed our second hypothesis. In line with recent studies, which indicate that higher education and being male is associated with positive vaccination intentions, this group had the lowest share of males and people with high education (27).

All groups experienced a reduction of fear between t1 and t2. However, the reduction of fear for people who were unvaccinated at t1 and received the vaccine between t1 and t2 (group 1) was significantly stronger than the fear reduction in all other groups, and in particular compared to the groups of refusers (group 3) and unvaccinated due to other reasons (group 4) (controlling for other factors, such as age, gender, education, income, and immigration status). This confirmed our third hypothesis. We interpret this as a positive effect of vaccination on the mental health condition of people who are concerned about the virus and not hesitant to get vaccinated. Considering that the peak of registered infections appeared in March 2021, the perceived threat of the virus and the need for a vaccine may have led many—especially those who felt vulnerable—to consider getting vaccinated as soon as they had the opportunity. Getting vaccinated appears to have been at least a partial alleviation of the psychological distress caused by the pandemic.

Our study, however, has several limitations. First, it does not allow us to answer whether refusers would have experienced the same decrease, had they been vaccinated. Thus, we cannot tell if the fear-reducing effect of vaccination would have operated also on the refusers (group 3). Second, those who began with a higher level of fear (i.e., group 1—the vaccinated and group 4—people unvaccinated for other reasons) had a higher potential to experience a stronger reduction of fear compared to those who began with lower levels of fear (i.e., refusers) due to the so-called floor effect. However, this floor effect might have a limited impact given that we also observed a similar reduction in fear for the group with the lowest initial fear level (group 3) and the one with the highest initial fear level (group 4). Third, due to the sampling procedure and the use of quotas, we are reluctant to generalize our findings to the general population of Germany. However, the sociodemographic sample characteristics as well as the vaccination rates in the sample were similar to those of the official statistics of the German population for the time of the study, suggesting that the data represent the population reliably. Fourth, drawing causal inferences from the results also relies on the assumption that, in the absence of vaccination, the level of fear would have developed in the same way across groups (i.e., the common trends assumption) (17, 28). Testing this assumption requires at least one additional measurement occasion prior to t1 or field experimental conditions that are not possible to design, because it is not possible to randomly exclude individuals from the possibility to receive a vaccination. Fifth, and finally, many potential factors that may influence the general level of fear of COVID-19 or differences in fear between groups and time could not be controlled (such as fear of vaccination or stable personality characteristics).

Yet, and having these limitations in mind, the design of the study and our findings suggest that fear of COVID-19 is not only a driver of the decision to get vaccinated (29), but also that the vaccination effectively reduces fear beyond the general trend. Thus, this study supports the notion that vaccine development, deployment, and promotion programs are one of the most efficient societal investments in sciences and technologies (30). In public health communication we recommend that scientists, practitioners, and politicians highlight the positive effect of vaccination against COVID-19 in addition to protection against serious illness, hospitalization, and death. In addition, the policy implications resulting from our findings may be relevant beyond understanding the past and current situation in Germany but also for future occurrences. We hope that our findings enable societies and policy makers to better understand the modus operandi of response strategies of individuals, to promote effective vaccines, and to enhance the willingness to get vaccinated by underlining that vaccines can reduce fear.

## Transparency statement

The authors affirm that the manuscript is an honest, accurate, and transparent account of the study being reported. No important aspects of the study have been omitted.

## Data availability statement

The data used in this study can be obtained from DS upon request. Anonymous individual participant data and a data dictionary can be obtained from the corresponding author upon request. Analytical codes are available in the Supplementary material submitted with the manuscript.

## Ethics statement

The studies involving human participants were reviewed and approved by Ethics Committee of the Faculty of Management, Economics and Social Sciences, and University of Cologne (Reference Numbers: 210005DS and 210031DS). Written informed consent for participation was not required for this study in accordance with the national legislation and the institutional requirements.

## Author contributions

DS: study and questionnaire design, data preparation, analysis, interpretation of results, writing, and proofreading. DM and ED: study and questionnaire design, interpretation of results, writing, and proofreading. All authors had access to and verified the validity of the data and results, contributed to the article, and approved the submitted version.

## Funding

The Fritz Thyssen Foundation generously provided funds for data collection (Grant Number: Az. 20.21.0.004SO). The funding organization had no influence on the study design, data collection, analysis, interpretation, or writing of the manuscript.

## Conflict of interest

The authors declare that the research was conducted in the absence of any commercial or financial relationships that could be construed as a potential conflict of interest.

## Publisher's note

All claims expressed in this article are solely those of the authors and do not necessarily represent those of their affiliated organizations, or those of the publisher, the editors and the reviewers. Any product that may be evaluated in this article, or claim that may be made by its manufacturer, is not guaranteed or endorsed by the publisher.

## References

[1] Quadros S Garg S Ranjan R Vijayasarathi G Mamun M. Fear of COVID 19 infection across different cohorts: a scoping review. Front Psychiatry. (2021) 12:708430. 10.3389/fpsyt.2021.708430 34557117 PMC8453018

[2] Luo F Ghanei Gheshlagh R Dalvand S Saedmoucheshi S Li Q. Systematic review and meta-analysis of fear of COVID-19. Front Psychol. (2021) 12:661078. 10.3389/fpsyg.2021.661078 34177712 PMC8231929

[3] Rodríguez-Hidalgo A Pantaleón Y Dios I Falla D. Fear of COVID-19, Stress, and anxiety in University undergraduate students: a predictive model for depression. Front Psychol. (2020) 11:591797. 10.3389/fpsyg.2020.591797 33224080 PMC7674167

[4] Belen H. Fear of COVID-19 and mental health: the role of mindfulness in during times of crisis. Int J Ment Health Addict. (2022). 20:607–18. 10.1007/s11469-020-00470-2 33935608 PMC8075278

[5] Iacob C Ionescu D Avram E Cojocaru D. COVID-19 pandemic worry and vaccination intention: the mediating role of the health belief model components. Front Psychol. (2021). 12:674018. 10.3389/fpsyg.2021.674018 PMC8311124 34322062

[6] Scrima F Miceli S Caci B Cardaci M. The relationship between fear of COVID-19 and intention to get vaccinated. The serial mediation roles of existential anxiety and conspiracy beliefs. Pers Individ Dif. (2021) 184:111188. 10.1016/j.paid.2021.111188 34393312 PMC8354796

[7] Bendau A Plag J Petzold M Ströhle A. COVID-19 vaccine hesitancy and related fears and anxiety. Int Immunopharmacol. (2021) 97:107724. 10.1016/j.intimp.2021.107724 33951558 PMC8078903

[8] Ordinance on the Entitlement to Vaccination Against the SARS-CoV-2 Coronavirus. Available online at: https://www.bundesgesundheitsministerium.de/fileadmin/Dateien/3_Downloads/C/Coronavirus/Verordnungen/CoronaImpfV_EN_080221.pdf. (accessed December 5, 2021).

[9] Vuong Q Le T La V Nguyen H Ho M Van Khuc Q et al. Covid-19 vaccines production and societal immunization under the serendipity-mindsponge-3D knowledge management theory and conceptual framework. Hum Soc Sci Commun. (2022) 9:22. 10.1057/s41599-022-01034-6

[10] Zhang J Wu W Zhao X Zhang W. Recommended psychological crisis intervention response to the 2019 novel coronavirus pneumonia outbreak in China: a model of West China Hospital. Precis Clin Med. (2020) 3:3–8. 10.1093/pcmedi/pbaa006 PMC7107095 35960676

[11] Perez-Arce F Angrisani M Bennett D Darling J Kapteyn A Thomas K. COVID-19 vaccines and mental distress. PLoS ONE. (2021) 16:e0256406. 10.1371/journal.pone.0256406 34496006 PMC8425550

[12] Bilge Y Keles E Baydili K. The impact of COVID-19 vaccination on mental health. J Loss Trauma. (2021) 27:1–4. 10.1080/15325024.2021.196355833552172

[13] Koltai J Raifman J Bor J McKee M Stuckler D. COVID-19 vaccination and mental health: a difference-in-difference analysis of the understanding America study. Am J Prev Med. (2021) 62:679–87. 10.1101/2021.07.19.21260782 35012830 PMC8674498

[14] respondiAG. Available online at: https://www.respondi.com/EN/ (accessed December 5, 2021).

[15] German Federal Statistical Office (Destatis),. Database of the Federal Statistical Office of Germany (GENESIS-Online). (2021). Available online at: https://www-genesis.destatis.de/genesis/online (accessed July 14, 2022).

[16] Impfdashboard.de. Available online at: https://impfdashboard.de/en/ (accessed December 5, 2021).

[17] Wing C Simon K Bello-Gomez R. Designing difference in difference studies: best practices for public health policy research. Annu Rev Public Health. (2018) 39:453–69. 10.1146/annurev-publhealth-040617-013507 29328877

[18] Ahorsu D Lin C Imani V Saffari M Griffiths M Pakpour A. The Fear of COVID-19 scale: development and initial validation. Int J Ment Health Addict. (2022) 20:1537–45. 10.1007/s11469-020-00270-8 32226353 PMC7100496

[19] Brown T. Confirmatory Factor Analysis for Applied Research. 2nd ed. New York: The Guilford Press (2015).

[20] Pentz M Chou C. Measurement invariance in longitudinal clinical research assuming change from development and intervention. J Consult Clin Psychol. (1994) 62:450–62. 10.1037/0022-006X.62.3.450 8063972

[21] Hancock G. Experimental, quasi-experimental, and nonexperimental design and analysis with latent variables. In: Kaplan D, editor. The SAGE Handbook of Quantitative Methodology for the Social Sciences. Thousand Oaks: Sage (2004). p. 317–34.

[22] Bollen K. Structural Equations With Latent Variables. New York, NY: Wiley (1989).

[23] West SG Taylor AB Wu W. Model fit and model selection in structural equation modeling. In: Rick H, editor. Handbook of Structural Equation Modeling. New York, NY: The Guilford Press (2012). p. 209–31.

[24] Rosseel Y. lavaan: An R Package for structural equation modeling. J Stat Soft. (2012) 48:1–36. 10.18637/jss.v048.i0225601849

[25] Robert Koch Institute. Available online at: https://www.rki.de/EN/Content/infections/epidemiology/outbreaks/COVID-19/COVID19.html (accessed December 5, 2021).

[26] Crawshaw A Farah Y Deal A Rustage K Hayward S Carter J. Defining the determinants of vaccine uptake and undervaccination in migrant populations in Europe to improve routine and COVID-19 vaccine uptake: a systematic review. Lancet Infect Dis. (2022). 10.1016/S1473-3099(22)00066-4 [Epub ahead of print]. PMC9007555 35429463

[27] Troiano G Nardi A. Vaccine hesitancy in the era of COVID-19. Public Health. (2021) 194:245–51. 10.1016/j.puhe.2021.02.025 33965796 PMC7931735

[28] Callaway B. Sant'Anna P. Difference-in-Differences with multiple time periods. J Econ. (2021) 225:200–30. 10.1016/j.jeconom.2020.12.001

[29] Seddig D Maskileyson D Davidov E Ajzen I Schmidt P. Correlates of COVID-19 vaccination intentions: attitudes, institutional trust, fear, conspiracy beliefs, and vaccine skepticism. Soc Sci Med. (2022) 302:114981. 10.1016/j.socscimed.2022.114981 35512613 PMC9017059

[30] Vuong QH. The (ir)rational consideration of the cost of science in transition economies. Nat Hum Behav. (2018) 2:5. 10.1038/s41562-017-0281-4 30980055

