# Supplementary material for: Vaccination against COVID-19 reduces virus-related fears: Findings from a German longitudinal study
Source: Front Public Health. 2022 Jul 28;10:878787. doi: 10.3389/fpubh.2022.878787 (PMC9366712; doi:10.3389/fpubh.2022.878787)
Supplement: Supplementary file 1 [file Data_Sheet_1.pdf]

Seddig, D., Maskileyson, D., & Davidov, E. (2022). Vaccination against COVID-19 reduces virus-related fears. Findings from a German longitudinal study. *Frontiers in Public Health*.

Supplementary Materials

Table S1. Descriptive statistics for the indicators measuring fear of COVID-19.

	N	Mean	Std. Dev.	Min	Max	Skewness	Kurtosis
Group 1: vaccinated between t_1 and t_2							
$fear_{11}$	2128	4.805	1.753	1	7	-0.577	-0.549
$fear_{21}$	2127	5.434	1.657	1	7	-1.061	0.349
$fear_{31}$	2127	3.827	1.845	1	7	0.045	-0.998
$fear_{12}$	2135	4.290	1.869	1	7	-0.234	-1.038
$fear_{22}$	2135	4.896	1.836	1	7	-0.630	-0.673
$fear_{32}$	2132	3.445	1.777	1	7	0.239	-0.917
Group 2: vaccinated prior to t_1							
$fear_{11}$	683	4.845	1.689	1	7	-0.609	-0.448
$fear_{21}$	683	5.537	1.542	1	7	-1.155	0.824
$fear_{31}$	683	3.865	1.749	1	7	-0.032	-0.912
$fear_{12}$	682	4.521	1.792	1	7	-0.396	-0.839
$fear_{22}$	682	5.129	1.715	1	7	-0.807	-0.264
$fear_{32}$	680	3.554	1.731	1	7	0.173	-0.887
Group 3: refusers							
$fear_{11}$	394	3.145	2.051	1	7	0.549	-0.961
$fear_{21}$	395	3.372	2.099	1	7	0.396	-1.135
$fear_{31}$	395	2.380	1.679	1	7	0.922	-0.267
$fear_{12}$	392	2.893	1.997	1	7	0.751	-0.669
$fear_{22}$	393	2.959	1.941	1	7	0.652	-0.725
$fear_{32}$	393	2.201	1.662	1	7	1.269	0.560
Group 4: unvaccinated for other reasons							
$fear_{11}$	204	4.657	1.798	1	7	-0.465	-0.674
$fear_{21}$	205	5.132	1.776	1	7	-0.804	-0.382
$fear_{31}$	205	3.868	1.932	1	7	-0.009	-1.149
$fear_{12}$	202	4.248	1.863	1	7	-0.287	-1.004
$fear_{22}$	202	4.782	1.737	1	7	-0.555	-0.551
$fear_{32}$	202	3.827	1.886	1	7	-0.034	-1.044
Total sample							
$fear_{11}$	3409	4.612	1.857	1	7	-0.485	-0.784
$fear_{21}$	3410	5.198	1.825	1	7	-0.909	-0.188
$fear_{31}$	3410	3.670	1.872	1	7	0.097	-1.043
$fear_{12}$	3411	4.173	1.926	1	7	-0.188	-1.115
$fear_{22}$	3410	4.712	1.928	1	7	-0.534	-0.873
$fear_{32}$	3407	3.346	1.811	1	7	0.283	-0.953

Notes: the first indicator subscript refers to the substantive indicator; the second indicator subscript refers to the time point.

Annotated R code and output.

```
library(descr)
library(lavaan)
library(psych)
library(semTools)
options(width=160,max.print = 1000000)

################################################################################

################################################################################
# R-script for
# Seddig, D., Maskileysion, D., & Davidov, E. (2022). Vaccination against
# COVID-19 reduces virus-related fears. Findings from a German longitudinal
# study. Frontiers in Public Health.
################################################################################

# Set a working directory
setwd("C:/")
fear_data <- read.csv("data seddig_etal_2022 fear of COVID19.csv",na=".k")
attach(fear_data)
library(descr)
library(lavaan)
library(psych)
library(semTools)
options(width=160,max.print = 1000000)

>
> ################################################################################
>
> ##### 0 Descriptive statistics #####
> freq(groups,plot=F)
groups
      Frequency Percent
1          2139  62.398
2           683  19.924
```

```
3             399  11.639
4             207   6.039
Total        3428 100.000
>
> x1<-data.frame(agea,male1,hedu,lincome1,imm1,fear11,fear21,fear31,fear12,fear22,fear32)
> # By groups
> des1<-describeBy(x1,group=groups)
> print(des1,digits=3)

 Descriptive statistics by group
group: 1
         vars    n   mean     sd median trimmed    mad min max range   skew kurtosis    se
agea        1 2139 49.004 14.195     51  49.529 16.309  18  74    56 -0.285   -0.922 0.307
male1       2 2139  0.554  0.497      1   0.567  0.000   0   1     1 -0.217   -1.954 0.011
hedu        3 2139  0.240  0.427      0   0.175  0.000   0   1     1  1.218   -0.517 0.009
lincome1    4 2139  0.276  0.447      0   0.221  0.000   0   1     1  1.000   -1.001 0.010
imm1        5 2139  0.166  0.372      0   0.083  0.000   0   1     1  1.794    1.220 0.008
fear11      6 2128  4.805  1.753      5   4.963  1.483   1   7     6 -0.577   -0.549 0.038
fear21      7 2127  5.434  1.657      6   5.685  1.483   1   7     6 -1.061    0.349 0.036
fear31      8 2127  3.827  1.845      4   3.792  1.483   1   7     6  0.045   -0.998 0.040
fear12      9 2135  4.290  1.869      4   4.363  2.965   1   7     6 -0.234   -1.038 0.040
fear22     10 2135  4.896  1.836      5   5.081  1.483   1   7     6 -0.630   -0.673 0.040
fear32     11 2132  3.445  1.777      3   3.365  1.483   1   7     6  0.239   -0.917 0.038
----------------------------------------------------------------------------------------------------------
----------------
group: 2
         vars   n   mean     sd median trimmed    mad min max range   skew kurtosis    se
agea        1 683 52.963 14.954     56  53.978 16.309  18  74    56 -0.499   -0.889 0.572
male1       2 683  0.551  0.498      1   0.563  0.000   0   1     1 -0.203   -1.962 0.019
hedu        3 683  0.225  0.418      0   0.157  0.000   0   1     1  1.311   -0.282 0.016
lincome1    4 683  0.250  0.434      0   0.188  0.000   0   1     1  1.150   -0.679 0.017
imm1        5 683  0.133  0.340      0   0.042  0.000   0   1     1  2.154    2.643 0.013
fear11      6 683  4.845  1.689      5   5.000  1.483   1   7     6 -0.609   -0.448 0.065
fear21      7 683  5.537  1.542      6   5.779  1.483   1   7     6 -1.155    0.824 0.059
fear31      8 683  3.865  1.749      4   3.874  1.483   1   7     6 -0.032   -0.912 0.067
fear12      9 682  4.521  1.792      5   4.623  1.483   1   7     6 -0.396   -0.839 0.069
fear22     10 682  5.129  1.715      6   5.344  1.483   1   7     6 -0.807   -0.264 0.066
fear32     11 680  3.554  1.731      4   3.502  1.483   1   7     6  0.173   -0.887 0.066
```

```
------------------------------------------------------------------------------------------------------
----------------
group: 3
         vars   n   mean     sd median trimmed    mad min max range   skew kurtosis    se
agea        1 399 45.353 13.464     46  45.305 16.309  18  73    55 -0.016   -0.891 0.674
male1       2 399  0.469  0.500      0   0.461  0.000   0   1     1  0.125   -1.989 0.025
hedu        3 399  0.158  0.365      0   0.075  0.000   0   1     1  1.869    1.498 0.018
lincome1    4 399  0.401  0.491      0   0.377  0.000   0   1     1  0.402   -1.843 0.025
imm1        5 399  0.185  0.389      0   0.109  0.000   0   1     1  1.612    0.601 0.019
fear11      6 394  3.145  2.051      3   2.934  2.965   1   7     6  0.549   -0.961 0.103
fear21      7 395  3.372  2.099      3   3.218  2.965   1   7     6  0.396   -1.135 0.106
fear31      8 395  2.380  1.679      2   2.129  1.483   1   7     6  0.922   -0.267 0.084
fear12      9 392  2.893  1.997      2   2.631  1.483   1   7     6  0.751   -0.669 0.101
fear22     10 393  2.959  1.941      3   2.727  2.965   1   7     6  0.652   -0.725 0.098
fear32     11 393  2.201  1.662      1   1.889  0.000   1   7     6  1.269    0.560 0.084
------------------------------------------------------------------------------------------------------
----------------
group: 4
         vars   n   mean     sd median trimmed    mad min max range   skew kurtosis    se
agea        1 207 42.261 13.234     41  41.725 14.826  19  74    55  0.314   -0.854 0.920
male1       2 207  0.512  0.501      1   0.515  0.000   0   1     1 -0.048   -2.007 0.035
hedu        3 207  0.222  0.417      0   0.156  0.000   0   1     1  1.327   -0.241 0.029
lincome1    4 207  0.391  0.489      0   0.365  0.000   0   1     1  0.442   -1.813 0.034
imm1        5 207  0.246  0.432      0   0.186  0.000   0   1     1  1.169   -0.637 0.030
fear11      6 204  4.657  1.798      5   4.799  1.483   1   7     6 -0.465   -0.674 0.126
fear21      7 205  5.132  1.776      6   5.345  1.483   1   7     6 -0.804   -0.382 0.124
fear31      8 205  3.868  1.932      4   3.836  2.965   1   7     6 -0.009   -1.149 0.135
fear12      9 202  4.248  1.863      4   4.309  2.965   1   7     6 -0.287   -1.004 0.131
fear22     10 202  4.782  1.737      5   4.932  1.483   1   7     6 -0.555   -0.551 0.122
fear32     11 202  3.827  1.886      4   3.796  1.483   1   7     6 -0.034   -1.044 0.133
> # Total sample
> des2<-describe(x1)
> print(des2,digits=3)
         vars    n   mean     sd median trimmed    mad min max range   skew kurtosis    se
agea        1 3428 48.961 14.464     51  49.381 16.309  18  74    56 -0.232   -0.986 0.247
male1       2 3428  0.541  0.498      1   0.551  0.000   0   1     1 -0.164   -1.974 0.009
hedu        3 3428  0.226  0.419      0   0.158  0.000   0   1     1  1.307   -0.291 0.007
lincome1    4 3428  0.293  0.455      0   0.241  0.000   0   1     1  0.911   -1.170 0.008
```

```
imm1         5 3428  0.167  0.373      0   0.083  0.000   0   1     1  1.789    1.201 0.006
fear11       6 3409  4.612  1.857      5   4.759  1.483   1   7     6 -0.485   -0.784 0.032
fear21       7 3410  5.198  1.825      6   5.460  1.483   1   7     6 -0.909   -0.188 0.031
fear31       8 3410  3.670  1.872      4   3.612  1.483   1   7     6  0.097   -1.043 0.032
fear12       9 3411  4.173  1.926      4   4.217  2.965   1   7     6 -0.188   -1.115 0.033
fear22      10 3412  4.712  1.928      5   4.886  1.483   1   7     6 -0.534   -0.873 0.033
fear32      11 3407  3.346  1.811      3   3.243  1.483   1   7     6  0.283   -0.953 0.031

>
> ################################################################################
>
> ##### 1 Confirmatory factor analysis (CFA) ####
>
> ### CFA t1 & t2
> cfa <-'
+ fear1=~fear11+fear21+fear31
+ fear2=~fear12+fear22+fear32
+ fear11~0*1
+ fear12~0*1
+ fear1~1
+ fear2~1
+ fear11~~fear12
+ fear21~~fear22
+ fear31~~fear32'
> fcfa<-cfa(cfa,data=fear_data,missing="FIML",estimator="MLR")
Warnmeldung:
In lav_data_full(data = data, group = group, cluster = cluster,  :
  lavaan WARNING: some cases are empty and will be ignored:
  982 1236 2707
> summary(fcfa,standardized=TRUE,fit.measures=TRUE)
lavaan 0.6-9 ended normally after 60 iterations

  Estimator                                         ML
  Optimization method                           NLMINB
  Number of model parameters                        22

                                                  Used       Total
  Number of observations                          3425        3428
```

```
  Number of missing patterns                        12

Model Test User Model:
                                              Standard      Robust
  Test Statistic                                38.040      28.432
  Degrees of freedom                                 5           5
  P-value (Chi-square)                           0.000       0.000
  Scaling correction factor                                  1.338
       Yuan-Bentler correction (Mplus variant)

Model Test Baseline Model:

  Test statistic                             13953.765    8006.090
  Degrees of freedom                                15          15
  P-value                                        0.000       0.000
  Scaling correction factor                                  1.743

User Model versus Baseline Model:

  Comparative Fit Index (CFI)                    0.998       0.997
  Tucker-Lewis Index (TLI)                       0.993       0.991

  Robust Comparative Fit Index (CFI)                         0.998
  Robust Tucker-Lewis Index (TLI)                            0.993

Loglikelihood and Information Criteria:

  Loglikelihood user model (H0)             -34868.499  -34868.499
  Scaling correction factor                                  1.317
      for the MLR correction
  Loglikelihood unrestricted model (H1)     -34849.479  -34849.479
  Scaling correction factor                                  1.321
      for the MLR correction

  Akaike (AIC)                               69780.998   69780.998
  Bayesian (BIC)                             69916.053   69916.053
  Sample-size adjusted Bayesian (BIC)        69846.149   69846.149
```

```
Root Mean Square Error of Approximation:

  RMSEA                                          0.044       0.037
  90 Percent confidence interval - lower         0.032       0.026
  90 Percent confidence interval - upper         0.057       0.049
  P-value RMSEA <= 0.05                          0.755       0.965

  Robust RMSEA                                               0.043
  90 Percent confidence interval - lower                     0.028
  90 Percent confidence interval - upper                     0.059

Standardized Root Mean Square Residual:

  SRMR                                           0.012       0.012

Parameter Estimates:

  Standard errors                             Sandwich
  Information bread                           Observed
  Observed information based on                Hessian

Latent Variables:
                   Estimate  Std.Err  z-value  P(>|z|)   Std.lv  Std.all
  fear1 =~
    fear11            1.000                               1.640    0.883
    fear21            0.925    0.017   54.654    0.000    1.517    0.832
    fear31            0.842    0.018   46.900    0.000    1.380    0.740
  fear2 =~
    fear12            1.000                               1.754    0.911
    fear22            0.935    0.015   61.056    0.000    1.641    0.848
    fear32            0.760    0.015   52.256    0.000    1.334    0.742

Covariances:
                   Estimate  Std.Err  z-value  P(>|z|)   Std.lv  Std.all
 .fear11 ~~
   .fear12           -0.045    0.035   -1.301    0.193   -0.045   -0.065
 .fear21 ~~
   .fear22            0.481    0.034   14.218    0.000    0.481    0.463
```

```
 .fear31 ~~
   .fear32             0.637    0.037   17.128    0.000    0.637    0.422
  fear1 ~~
    fear2              2.342    0.065   35.819    0.000    0.814    0.814

Intercepts:
                   Estimate  Std.Err  z-value  P(>|z|)   Std.lv  Std.all
   .fear11             0.000                               0.000    0.000
   .fear12             0.000                               0.000    0.000
    fear1              4.610    0.032  145.054    0.000    2.811    2.811
    fear2              4.171    0.033  126.586    0.000    2.378    2.378
   .fear21             0.932    0.089   10.478    0.000    0.932    0.511
   .fear31            -0.209    0.084   -2.484    0.013   -0.209   -0.112
   .fear22             0.806    0.074   10.842    0.000    0.806    0.417
   .fear32             0.171    0.061    2.789    0.005    0.171    0.095

Variances:
                   Estimate  Std.Err  z-value  P(>|z|)   Std.lv  Std.all
   .fear11             0.760    0.064   11.862    0.000    0.760    0.220
   .fear21             1.026    0.042   24.438    0.000    1.026    0.308
   .fear31             1.575    0.050   31.314    0.000    1.575    0.453
   .fear12             0.633    0.060   10.615    0.000    0.633    0.171
   .fear22             1.050    0.047   22.315    0.000    1.050    0.281
   .fear32             1.449    0.049   29.679    0.000    1.449    0.449
    fear1              2.688    0.086   31.098    0.000    1.000    1.000
    fear2              3.077    0.080   38.530    0.000    1.000    1.000

> reliability(fcfa,return.total=TRUE)
           fear1     fear2     total
alpha  0.8575532 0.8733510 0.9109533
omega  0.8596237 0.8771382 0.9001578
omega2 0.8596237 0.8771382 0.9001578
omega3 0.8573024 0.8716860 0.8934250
avevar 0.6722696 0.7067400 0.6898564

>
> ################################################################################
>
```

```
> ##### 2 Measurement invariance (MI) across time, per group #####
>
> # Configural MI model
> mi1 <-'
+ fear1=~fear11+fear21+fear31
+ fear2=~fear12+fear22+fear32
+ fear11~0*1
+ fear12~0*1
+ fear1~1
+ fear2~1
+ fear11~~fear12
+ fear21~~fear22
+ fear31~~fear32'
>
> # Metric MI model
> mi2 <-'
+ fear1=~a*fear11+b*fear21+c*fear31
+ fear2=~a*fear12+b*fear22+c*fear32
+ fear11~0*1
+ fear12~0*1
+ fear1~1
+ fear2~1
+ fear11~~fear12
+ fear21~~fear22
+ fear31~~fear32'
>
> # Scalar MI model
> mi3 <-'
+ fear1=~a*fear11+b*fear21+c*fear31
+ fear2=~a*fear12+b*fear22+c*fear32
+ fear11~0*1
+ fear12~0*1
+ fear21~d*1
+ fear22~d*1
+ fear31~e*1
+ fear32~e*1
+ fear1~1
+ fear2~1
```

```
+ fear11~~fear12
+ fear21~~fear22
+ fear31~~fear32'
>
> ### Group 1: Vaccinated between t1 and t2
> # Configural MI
> fmi1<-cfa(mi1,data=subset(fear_data,groups=="1"),missing="FIML",estimator="MLR")
Warnmeldung:
In lav_data_full(data = data, group = group, cluster = cluster,  :
  lavaan WARNING: some cases are empty and will be ignored:
  640 800
> # Metric MI
> fmi2<-cfa(mi2,data=subset(fear_data,groups=="1"),missing="FIML",estimator="MLR")
Warnmeldung:
In lav_data_full(data = data, group = group, cluster = cluster,  :
  lavaan WARNING: some cases are empty and will be ignored:
  640 800
> # Scalar MI
> fmi3<-cfa(mi3,data=subset(fear_data,groups=="1"),missing="FIML",estimator="MLR")
Warnmeldung:
In lav_data_full(data = data, group = group, cluster = cluster,  :
  lavaan WARNING: some cases are empty and will be ignored:
  640 800
> # Fit comparison
> c1<-compareFit(fmi1,fmi2,fmi3,nested=TRUE,indices=TRUE)
> c1
################### Nested Model Comparison #########################
Scaled Chi-Squared Difference Test (method = “satorra.bentler.2001”)

lavaan NOTE:
    The “Chisq” column contains standard test statistics, not the
    robust test that should be reported per model. A robust difference
    test is a function of two standard (not robust) statistics.

     Df   AIC   BIC  Chisq Chisq diff Df diff Pr(>Chisq)
fmi1  5 43146 43271 40.348
fmi2  7 43167 43280 65.099    26.7313       2  1.568e-06 ***
fmi3  9 43173 43275 74.885     9.7599       2   0.007597 **
```

```
---
Signif. codes:  0 ‘***’ 0.001 ‘**’ 0.01 ‘*’ 0.05 ‘.’ 0.1 ‘ ’ 1

####################### Model Fit Indices ###########################
     chisq.scaled df.scaled pvalue.scaled cfi.robust tli.robust        aic        bic rmsea.robust  srmr
fmi1      30.697†         5          .000     0.996†      .987† 43146.154† 43270.832†        .056† .017†
fmi2      54.095          7          .000      .993       .985  43166.905  43280.248         .062  .032
fmi3      64.622          9          .000      .992       .987  43172.692  43274.701         .058  .034

################## Differences in Fit Indices #######################
          df.scaled cfi.robust tli.robust    aic    bic rmsea.robust  srmr
fmi2 - fmi1         2     -0.003     -0.002 20.750  9.416        0.005 0.015
fmi3 - fmi2         2     -0.001      0.002  5.787 -5.547       -0.004 0.002

>
> ### Group 2: Vaccinated before t1
> # Configural MI
> fmi4<-cfa(mi1,data=subset(fear_data,groups=="2"),missing="FIML",estimator="MLR")
> # Metric MI
> fmi5<-cfa(mi2,data=subset(fear_data,groups=="2"),missing="FIML",estimator="MLR")
> # Scalar MI
> fmi6<-cfa(mi3,data=subset(fear_data,groups=="2"),missing="FIML",estimator="MLR")
> # Fit comparison
> c2<-compareFit(fmi4,fmi5,fmi6,nested=TRUE,indices=TRUE)
> c2
################### Nested Model Comparison #########################
Scaled Chi-Squared Difference Test (method = “satorra.bentler.2001”)

lavaan NOTE:
    The “Chisq” column contains standard test statistics, not the
    robust test that should be reported per model. A robust difference
    test is a function of two standard (not robust) statistics.

     Df   AIC   BIC   Chisq Chisq diff Df diff Pr(>Chisq)
fmi4  5 13583 13682  4.2871
fmi5  7 13596 13687 22.0601    19.6520       2  5.403e-05 ***
fmi6  9 13601 13682 30.2834     7.9742       2    0.01855 *
---
```

```
Signif. codes:  0 ‘***’ 0.001 ‘**’ 0.01 ‘*’ 0.05 ‘.’ 0.1 ‘ ’ 1

####################### Model Fit Indices ###########################
     chisq.scaled df.scaled pvalue.scaled cfi.robust tli.robust        aic        bic rmsea.robust  srmr
fmi4       3.151†         5          .677     1.000†     1.003† 13582.651† 13682.233         .000† .010†
fmi5      17.933          7          .012      .994       .988  13596.424  13686.953         .053  .036
fmi6      25.535          9          .002      .992       .986  13600.647  13682.124†        .056  .037

################## Differences in Fit Indices #######################
            df.scaled cfi.robust tli.robust    aic   bic rmsea.robust  srmr
fmi5 - fmi4         2     -0.006     -0.015 13.773  4.72        0.053 0.025
fmi6 - fmi5         2     -0.003     -0.002  4.223 -4.83        0.003 0.001

>
> ### Group 3: Not vaccinated (refusal)
> # Configural MI
> fmi7<-cfa(mi1,data=subset(fear_data,groups=="3"),missing="FIML",estimator="MLR")
Warnmeldung:
In lav_data_full(data = data, group = group, cluster = cluster,  :
  lavaan WARNING: some cases are empty and will be ignored:
  313
> # Metric MI
> fmi8<-cfa(mi2,data=subset(fear_data,groups=="3"),missing="FIML",estimator="MLR")
Warnmeldung:
In lav_data_full(data = data, group = group, cluster = cluster,  :
  lavaan WARNING: some cases are empty and will be ignored:
  313
> # Scalar MI
> fmi9<-cfa(mi3,data=subset(fear_data,groups=="3"),missing="FIML",estimator="MLR")
Warnmeldung:
In lav_data_full(data = data, group = group, cluster = cluster,  :
  lavaan WARNING: some cases are empty and will be ignored:
  313
> # Fit comparison
> c3<-compareFit(fmi7,fmi8,fmi9,nested=TRUE,indices=TRUE)
> c3
################### Nested Model Comparison #########################
Scaled Chi-Squared Difference Test (method = “satorra.bentler.2001”)
```

```
lavaan NOTE:
    The “Chisq” column contains standard test statistics, not the
    robust test that should be reported per model. A robust difference
    test is a function of two standard (not robust) statistics.

     Df    AIC    BIC   Chisq Chisq diff Df diff Pr(>Chisq)
fmi7  5 8113.0 8200.7  4.8178
fmi8  7 8110.4 8190.1  6.2699     1.5452       2     0.4618
fmi9  9 8110.4 8182.1 10.2363     3.9053       2     0.1419

####################### Model Fit Indices ###########################
     chisq.scaled df.scaled pvalue.scaled cfi.robust tli.robust       aic       bic rmsea.robust  srmr
fmi7       3.528†         5          .619     1.000†     1.004† 8112.965  8200.667          .000† .009†
fmi8       5.040          7          .655     1.000†     1.003  8110.417  8190.146          .000† .014
fmi9       8.579          9          .477     1.000†     1.001  8110.384† 8182.140†         .000† .017

################## Differences in Fit Indices #######################
            df.scaled cfi.robust tli.robust    aic     bic rmsea.robust  srmr
fmi8 - fmi7         2          0      0.000 -2.548 -10.521            0 0.006
fmi9 - fmi8         2          0     -0.003 -0.034  -8.007            0 0.003

>
> ### Group 4: Not vaccinated (other reasons)
> # Configural MI
> fmi10<-cfa(mi1,data=subset(fear_data,groups=="4"),missing="FIML",estimator="MLR")
> # Metric MI
> fmi11<-cfa(mi2,data=subset(fear_data,groups=="4"),missing="FIML",estimator="MLR")
> # Scalar MI
> fmi12<-cfa(mi3,data=subset(fear_data,groups=="4"),missing="FIML",estimator="MLR")
> # Fit comparison
> c4<-compareFit(fmi10,fmi11,fmi12,nested=TRUE,indices=TRUE)
> c4
################### Nested Model Comparison #########################
Scaled Chi-Squared Difference Test (method = “satorra.bentler.2001”)

lavaan NOTE:
    The “Chisq” column contains standard test statistics, not the
```

```
    robust test that should be reported per model. A robust difference
    test is a function of two standard (not robust) statistics.

      Df    AIC    BIC  Chisq Chisq diff Df diff Pr(>Chisq)
fmi10  5 4248.8 4322.2 12.869
fmi11  7 4246.1 4312.8 14.150     1.2973       2    0.52276
fmi12  9 4250.0 4309.9 21.995     8.5763       2    0.01373 *
---
Signif. codes:  0 '***' 0.001 '**' 0.01 '*' 0.05 '.' 0.1 ' ' 1

####################### Model Fit Indices ###########################
      chisq.scaled df.scaled pvalue.scaled cfi.robust tli.robust       aic       bic rmsea.robust  srmr
fmi10       9.965†         5          .076      .991       .973  4248.832  4322.152         .079  .022†
fmi11      11.747          7          .109      .992†      .983† 4246.113† 4312.767         .063† .026
fmi12      19.291          9          .023      .984       .973  4249.957  4309.946†        .079  .036

################## Differences in Fit Indices #######################
              df.scaled cfi.robust tli.robust    aic    bic rmsea.robust  srmr
fmi11 - fmi10         2      0.001       0.01 -2.719 -9.385       -0.016 0.004
fmi12 - fmi11         2     -0.008      -0.01  3.845 -2.821        0.017 0.010

>
> ################################################################################
>
> ##### 3 Measurement invariance (MI) across groups                      #####
> ##### (scalar MI was specified across time for all subsequent models) #####
>
> # Configural MI across groups
> mi4 <-'
+ group: [1]
+ fear1=~1*fear11+a*fear21+b*fear31
+ fear2=~1*fear12+a*fear22+b*fear32
+ fear11~0*1
+ fear12~0*1
+ fear21~c*1
+ fear22~c*1
+ fear31~d*1
+ fear32~d*1
```

```
+ fear1~1
+ fear2~1
+ fear11~~fear12
+ fear21~~fear22
+ fear31~~fear32
+ group: [2]
+ fear1=~1*fear11+e*fear21+f*fear31
+ fear2=~1*fear12+e*fear22+f*fear32
+ fear11~0*1
+ fear12~0*1
+ fear21~g*1
+ fear22~g*1
+ fear31~h*1
+ fear32~h*1
+ fear1~1
+ fear2~1
+ fear11~~fear12
+ fear21~~fear22
+ fear31~~fear32
+ group: [3]
+ fear1=~1*fear11+i*fear21+j*fear31
+ fear2=~1*fear12+i*fear22+j*fear32
+ fear11~0*1
+ fear12~0*1
+ fear21~k*1
+ fear22~k*1
+ fear31~l*1
+ fear32~l*1
+ fear1~1
+ fear2~1
+ fear11~~fear12
+ fear21~~fear22
+ fear31~~fear32
+ group: [4]
+ fear1=~1*fear11+m*fear21+n*fear31
+ fear2=~1*fear12+m*fear22+n*fear32
+ fear11~0*1
+ fear12~0*1
```

```
+ fear21~o*1
+ fear22~o*1
+ fear31~p*1
+ fear32~p*1
+ fear1~1
+ fear2~1
+ fear11~~fear12
+ fear21~~fear22
+ fear31~~fear32
+ '
> fmi13<-cfa(mi4,data=fear_data,missing="FIML",estimator="MLR",group="groups")
Warnmeldungen:
1: In lav_data_full(data = data, group = group, cluster = cluster,  :
  lavaan WARNING: some cases are empty and will be ignored:
  982 1236
2: In lav_data_full(data = data, group = group, cluster = cluster,  :
  lavaan WARNING: some cases are empty and will be ignored:
  2707
>
> # Metric MI across groups
> mi5 <-'
+ group: [1]
+ fear1=~1*fear11+a*fear21+b*fear31
+ fear2=~1*fear12+a*fear22+b*fear32
+ fear11~0*1
+ fear12~0*1
+ fear21~c*1
+ fear22~c*1
+ fear31~d*1
+ fear32~d*1
+ fear1~1
+ fear2~1
+ fear11~~fear12
+ fear21~~fear22
+ fear31~~fear32
+ group: [2]
+ fear1=~1*fear11+a*fear21+b*fear31
+ fear2=~1*fear12+a*fear22+b*fear32
```

```
+ fear11~0*1
+ fear12~0*1
+ fear21~g*1
+ fear22~g*1
+ fear31~h*1
+ fear32~h*1
+ fear1~1
+ fear2~1
+ fear11~~fear12
+ fear21~~fear22
+ fear31~~fear32
+ group: [3]
+ fear1=~1*fear11+a*fear21+b*fear31
+ fear2=~1*fear12+a*fear22+b*fear32
+ fear11~0*1
+ fear12~0*1
+ fear21~k*1
+ fear22~k*1
+ fear31~l*1
+ fear32~l*1
+ fear1~1
+ fear2~1
+ fear11~~fear12
+ fear21~~fear22
+ fear31~~fear32
+ group: [4]
+ fear1=~1*fear11+a*fear21+b*fear31
+ fear2=~1*fear12+a*fear22+b*fear32
+ fear11~0*1
+ fear12~0*1
+ fear21~o*1
+ fear22~o*1
+ fear31~p*1
+ fear32~p*1
+ fear1~1
+ fear2~1
+ fear11~~fear12
+ fear21~~fear22
```

```
+ fear31~~fear32
+ '
> fmi14<-cfa(mi5,data=fear_data,missing="FIML",estimator="MLR",group="groups")
Warnmeldungen:
1: In lav_data_full(data = data, group = group, cluster = cluster,  :
  lavaan WARNING: some cases are empty and will be ignored:
  982 1236
2: In lav_data_full(data = data, group = group, cluster = cluster,  :
  lavaan WARNING: some cases are empty and will be ignored:
  2707
>
> # Scalar MI across groups
> mi6 <-'
+ group: [1]
+ fear1=~1*fear11+a*fear21+b*fear31
+ fear2=~1*fear12+a*fear22+b*fear32
+ fear11~0*1
+ fear12~0*1
+ fear21~c*1
+ fear22~c*1
+ fear31~d*1
+ fear32~d*1
+ fear1~g11*1
+ fear2~g12*1
+ fear11~~fear12
+ fear21~~fear22
+ fear31~~fear32
+ group: [2]
+ fear1=~1*fear11+a*fear21+b*fear31
+ fear2=~1*fear12+a*fear22+b*fear32
+ fear11~0*1
+ fear12~0*1
+ fear21~c*1
+ fear22~c*1
+ fear31~d*1
+ fear32~d*1
+ fear1~g21*1
+ fear2~g22*1
```

```
+ fear11~~fear12
+ fear21~~fear22
+ fear31~~fear32
+ group: [3]
+ fear1=~1*fear11+a*fear21+b*fear31
+ fear2=~1*fear12+a*fear22+b*fear32
+ fear11~0*1
+ fear12~0*1
+ fear21~c*1
+ fear22~c*1
+ fear31~d*1
+ fear32~d*1
+ fear1~g31*1
+ fear2~g32*1
+ fear11~~fear12
+ fear21~~fear22
+ fear31~~fear32
+ group: [4]
+ fear1=~1*fear11+a*fear21+b*fear31
+ fear2=~1*fear12+a*fear22+b*fear32
+ fear11~0*1
+ fear12~0*1
+ fear21~c*1
+ fear22~c*1
+ fear31~d*1
+ fear32~d*1
+ fear1~g41*1
+ fear2~g42*1
+ fear11~~fear12
+ fear21~~fear22
+ fear31~~fear32
+ '
> fmi15<-cfa(mi6,data=fear_data,missing="FIML",estimator="MLR",group="groups")
Warnmeldungen:
1: In lav_data_full(data = data, group = group, cluster = cluster,  :
  lavaan WARNING: some cases are empty and will be ignored:
  982 1236
2: In lav_data_full(data = data, group = group, cluster = cluster,  :
```

```
  lavaan WARNING: some cases are empty and will be ignored:
  2707
> c5<-compareFit(fmi13,fmi14,fmi15,nested=TRUE,indices=TRUE)
> c5
################### Nested Model Comparison #########################
Scaled Chi-Squared Difference Test (method = “satorra.bentler.2001”)

lavaan NOTE:
    The “Chisq” column contains standard test statistics, not the
    robust test that should be reported per model. A robust difference
    test is a function of two standard (not robust) statistics.

      Df   AIC   BIC  Chisq Chisq diff Df diff Pr(>Chisq)
fmi13 36 69134 69576 137.40
fmi14 42 69161 69567 177.14     35.015       6   4.28e-06 ***
fmi15 48 69259 69628 287.02    101.130       6  < 2.2e-16 ***
---
Signif. codes:  0 ‘***’ 0.001 ‘**’ 0.01 ‘*’ 0.05 ‘.’ 0.1 ‘ ’ 1

####################### Model Fit Indices ###########################
      chisq.scaled df.scaled pvalue.scaled cfi.robust tli.robust        aic        bic rmsea.robust  srmr
fmi13     117.484†        36          .000      .993†      .988† 69133.680† 69575.677         .056† .033†
fmi14     152.107         42          .000      .990       .986  69161.423  69566.588†        .060  .041
fmi15     248.539         48          .000      .982       .977  69259.301  69627.633         .075  .048

################## Differences in Fit Indices #######################
              df.scaled cfi.robust tli.robust    aic    bic rmsea.robust  srmr
fmi14 - fmi13         6     -0.003     -0.002 27.744 -9.089        0.004 0.009
fmi15 - fmi14         6     -0.008     -0.008 97.878 61.045        0.015 0.007

>
> ################################################################################
>
> ##### 4 Final model estimating differences-in-differences (DiD) #####
> #####   and controlling for age, gender, education, low income, #####
> #####   and immigrant status                                    #####
>
> sem1 <-'
```

```
+ group: [1]
+ fear1=~1*fear11+a*fear21+b*fear31
+ fear2=~1*fear12+a*fear22+b*fear32
+ fear11~0*1
+ fear12~0*1
+ fear21~c*1
+ fear22~c*1
+ fear31~d*1
+ fear32~d*1
+ fear1~g11*1
+ fear2~g12*1
+ fear11~~fear12
+ fear21~~fear22
+ fear31~~fear32
+ # control
+ fear1~h1*ageb+h2*male1+h3*hedu+h4*lincome1+h5*imm1
+ fear2~h1*ageb+h2*male1+h3*hedu+h4*lincome1+h5*imm1
+ group: [2]
+ fear1=~1*fear11+a*fear21+b*fear31
+ fear2=~1*fear12+a*fear22+b*fear32
+ fear11~0*1
+ fear12~0*1
+ fear21~c*1
+ fear22~c*1
+ fear31~d*1
+ fear32~d*1
+ fear1~g21*1
+ fear2~g22*1
+ fear11~~fear12
+ fear21~~fear22
+ fear31~~fear32
+ # control
+ fear1~i1*ageb+i2*male1+i3*hedu+i4*lincome1+i5*imm1
+ fear2~i1*ageb+i2*male1+i3*hedu+i4*lincome1+i5*imm1
+ group: [3]
+ fear1=~1*fear11+a*fear21+b*fear31
+ fear2=~1*fear12+a*fear22+b*fear32
+ fear11~0*1
```

```
+ fear12~0*1
+ fear21~c*1
+ fear22~c*1
+ fear31~d*1
+ fear32~d*1
+ fear1~g31*1
+ fear2~g32*1
+ fear11~~fear12
+ fear21~~fear22
+ fear31~~fear32
+ # control
+ fear1~j1*ageb+j2*male1+j3*hedu+j4*lincome1+j5*imm1
+ fear2~j1*ageb+j2*male1+j3*hedu+j4*lincome1+j5*imm1
+ group: [4]
+ fear1=~1*fear11+a*fear21+b*fear31
+ fear2=~1*fear12+a*fear22+b*fear32
+ fear11~0*1
+ fear12~0*1
+ fear21~c*1
+ fear22~c*1
+ fear31~d*1
+ fear32~d*1
+ fear1~g41*1
+ fear2~g42*1
+ fear11~~fear12
+ fear21~~fear22
+ fear31~~fear32
+ # control
+ fear1~k1*ageb+k2*male1+k3*hedu+k4*lincome1+k5*imm1
+ fear2~k1*ageb+k2*male1+k3*hedu+k4*lincome1+k5*imm1
+ ### latent mean differences-in-differences
+ # group 1 vs group 2
+ did12 := (g12-g11)-(g22-g21)
+ # group 1 vs group 3
+ did13 := (g12-g11)-(g32-g31)
+ # group 1 vs group 4
+ did14 := (g12-g11)-(g42-g41)
+ # group 2 vs group 3
```

```
+ did23 := (g22-g21)-(g32-g31)
+ # group 2 vs group 4
+ did24 := (g22-g21)-(g42-g41)
+ # group 3 vs group 4
+ did34 := (g32-g31)-(g42-g41)
+ '
> fsem1<-sem(sem1,data=fear_data,missing="FIML",estimator="MLR",group="groups")
> summary(fsem1,standardized=TRUE,fit.measures=TRUE,ci=TRUE)
lavaan 0.6-9 ended normally after 255 iterations

  Estimator                                         ML
  Optimization method                           NLMINB
  Number of model parameters                       128
  Number of equality constraints                    48

  Number of observations per group:
    1                                             2139
    2                                              683
    3                                              399
    4                                              207
  Number of missing patterns per group:
    1                                               10
    2                                                3
    3                                               10
    4                                                6

Model Test User Model:
                                               Standard      Robust
  Test Statistic                                464.924     442.869
  Degrees of freedom                                148         148
  P-value (Chi-square)                            0.000       0.000
  Scaling correction factor                                   1.050
       Yuan-Bentler correction (Mplus variant)
  Test statistic for each group:
    1                                           156.680     149.248
    2                                            96.566      91.985
    3                                           134.748     128.356
    4                                            76.929      73.280
```

```
Model Test Baseline Model:

  Test statistic                             13123.533   10494.548
  Degrees of freedom                               180         180
  P-value                                        0.000       0.000
  Scaling correction factor                                  1.251

User Model versus Baseline Model:

  Comparative Fit Index (CFI)                    0.976       0.971
  Tucker-Lewis Index (TLI)                       0.970       0.965

  Robust Comparative Fit Index (CFI)                         0.976
  Robust Tucker-Lewis Index (TLI)                            0.971

Loglikelihood and Information Criteria:

  Loglikelihood user model (H0)             -34542.960  -34542.960
  Scaling correction factor                                  0.865
      for the MLR correction
  Loglikelihood unrestricted model (H1)     -34310.498  -34310.498
  Scaling correction factor                                  1.167
      for the MLR correction

  Akaike (AIC)                               69245.921   69245.921
  Bayesian (BIC)                             69737.099   69737.099
  Sample-size adjusted Bayesian (BIC)        69482.902   69482.902

Root Mean Square Error of Approximation:

  RMSEA                                          0.050       0.048
  90 Percent confidence interval - lower         0.045       0.043
  90 Percent confidence interval - upper         0.055       0.053
  P-value RMSEA <= 0.05                          0.492       0.712

  Robust RMSEA                                               0.049
  90 Percent confidence interval - lower                     0.044
```

```
  90 Percent confidence interval - upper                       0.055

Standardized Root Mean Square Residual:

  SRMR                                         0.034       0.034

Parameter Estimates:

  Standard errors                           Sandwich
  Information bread                         Observed
  Observed information based on              Hessian


Group 1 [1]:

Latent Variables:
                   Estimate  Std.Err  z-value  P(>|z|) ci.lower ci.upper   Std.lv  Std.all
  fear1 =~
    fear11            1.000                               1.000    1.000    1.508    0.862
    fear21     (a)    0.939    0.014   65.156    0.000    0.910    0.967    1.415    0.829
    fear31     (b)    0.788    0.012   66.529    0.000    0.765    0.811    1.188    0.668
  fear2 =~
    fear12            1.000                               1.000    1.000    1.678    0.911
    fear22     (a)    0.939    0.014   65.156    0.000    0.910    0.967    1.575    0.844
    fear32     (b)    0.788    0.012   66.529    0.000    0.765    0.811    1.323    0.745

Regressions:
                   Estimate  Std.Err  z-value  P(>|z|) ci.lower ci.upper   Std.lv  Std.all
  fear1 ~
    ageb      (h1)    0.238    0.078    3.050    0.002    0.085    0.392    0.158    0.070
    male1     (h2)   -0.247    0.069   -3.587    0.000   -0.381   -0.112   -0.164   -0.081
    hedu      (h3)    0.131    0.080    1.639    0.101   -0.026    0.289    0.087    0.037
    lincome1  (h4)    0.081    0.081    1.002    0.316   -0.077    0.239    0.054    0.024
    imm1      (h5)    0.136    0.088    1.542    0.123   -0.037    0.308    0.090    0.033
  fear2 ~
    ageb      (h1)    0.238    0.078    3.050    0.002    0.085    0.392    0.142    0.063
    male1     (h2)   -0.247    0.069   -3.587    0.000   -0.381   -0.112   -0.147   -0.073
    hedu      (h3)    0.131    0.080    1.639    0.101   -0.026    0.289    0.078    0.033
```

```
    lincome1   (h4)     0.081    0.081    1.002    0.316   -0.077    0.239    0.048    0.022
    imm1       (h5)     0.136    0.088    1.542    0.123   -0.037    0.308    0.081    0.030

Covariances:
                    Estimate  Std.Err  z-value  P(>|z|) ci.lower ci.upper   Std.lv  Std.all
 .fear11 ~~
   .fear12            -0.035    0.041   -0.855    0.393   -0.115    0.045   -0.035   -0.052
 .fear21 ~~
   .fear22             0.425    0.039   10.911    0.000    0.349    0.501    0.425    0.445
 .fear31 ~~
   .fear32             0.635    0.046   13.860    0.000    0.545    0.725    0.635    0.406
 .fear1 ~~
   .fear2              1.976    0.076   25.975    0.000    1.827    2.125    0.790    0.790

Intercepts:
                    Estimate  Std.Err  z-value  P(>|z|) ci.lower ci.upper   Std.lv  Std.all
   .fear11             0.000                               0.000    0.000    0.000    0.000
   .fear12             0.000                               0.000    0.000    0.000    0.000
   .fear21      (c)    0.856    0.073   11.693    0.000    0.713    0.999    0.856    0.501
   .fear22      (c)    0.856    0.073   11.693    0.000    0.713    0.999    0.856    0.459
   .fear31      (d)    0.061    0.050    1.205    0.228   -0.038    0.160    0.061    0.034
   .fear32      (d)    0.061    0.050    1.205    0.228   -0.038    0.160    0.061    0.034
   .fear1     (g11)    4.827    0.067   72.415    0.000    4.696    4.957    3.201    3.201
   .fear2     (g12)    4.287    0.068   62.637    0.000    4.153    4.421    2.555    2.555

Variances:
                    Estimate  Std.Err  z-value  P(>|z|) ci.lower ci.upper   Std.lv  Std.all
   .fear11             0.785    0.069   11.457    0.000    0.651    0.919    0.785    0.257
   .fear21             0.915    0.048   19.041    0.000    0.821    1.009    0.915    0.314
   .fear31             1.748    0.060   28.953    0.000    1.630    1.867    1.748    0.553
   .fear12             0.580    0.057   10.087    0.000    0.467    0.692    0.580    0.171
   .fear22             0.999    0.052   19.040    0.000    0.896    1.102    0.999    0.287
   .fear32             1.401    0.057   24.405    0.000    1.288    1.513    1.401    0.445
   .fear1              2.244    0.087   25.853    0.000    2.074    2.414    0.987    0.987
   .fear2              2.787    0.090   31.136    0.000    2.611    2.962    0.990    0.990


Group 2 [2]:
```

```
Latent Variables:
                   Estimate  Std.Err  z-value  P(>|z|) ci.lower ci.upper   Std.lv  Std.all
  fear1 =~
    fear11            1.000                               1.000    1.000    1.450    0.874
    fear21     (a)    0.939    0.014   65.156    0.000    0.910    0.967    1.361    0.831
    fear31     (b)    0.788    0.012   66.529    0.000    0.765    0.811    1.142    0.680
  fear2 =~
    fear12            1.000                               1.000    1.000    1.571    0.898
    fear22     (a)    0.939    0.014   65.156    0.000    0.910    0.967    1.475    0.840
    fear32     (b)    0.788    0.012   66.529    0.000    0.765    0.811    1.238    0.695

Regressions:
                   Estimate  Std.Err  z-value  P(>|z|) ci.lower ci.upper   Std.lv  Std.all
  fear1 ~
    ageb      (i1)    0.182    0.119    1.531    0.126   -0.051    0.415    0.126    0.062
    male1     (i2)   -0.062    0.117   -0.534    0.593   -0.292    0.167   -0.043   -0.021
    hedu      (i3)    0.054    0.142    0.378    0.705   -0.224    0.332    0.037    0.015
    lincome1  (i4)    0.238    0.135    1.762    0.078   -0.027    0.502    0.164    0.071
    imm1      (i5)   -0.051    0.146   -0.349    0.727   -0.337    0.235   -0.035   -0.012
  fear2 ~
    ageb      (i1)    0.182    0.119    1.531    0.126   -0.051    0.415    0.116    0.057
    male1     (i2)   -0.062    0.117   -0.534    0.593   -0.292    0.167   -0.040   -0.020
    hedu      (i3)    0.054    0.142    0.378    0.705   -0.224    0.332    0.034    0.014
    lincome1  (i4)    0.238    0.135    1.762    0.078   -0.027    0.502    0.151    0.066
    imm1      (i5)   -0.051    0.146   -0.349    0.727   -0.337    0.235   -0.032   -0.011

Covariances:
                   Estimate  Std.Err  z-value  P(>|z|) ci.lower ci.upper   Std.lv  Std.all
 .fear11 ~~
   .fear12            0.032    0.063    0.509    0.611   -0.092    0.156    0.032    0.052
 .fear21 ~~
   .fear22            0.347    0.066    5.296    0.000    0.219    0.475    0.347    0.400
 .fear31 ~~
   .fear32            0.654    0.077    8.478    0.000    0.503    0.805    0.654    0.415
 .fear1 ~~
   .fear2             1.717    0.129   13.277    0.000    1.464    1.971    0.761    0.761
```

```
Intercepts:
                   Estimate  Std.Err  z-value  P(>|z|) ci.lower ci.upper   Std.lv  Std.all
   .fear11            0.000                               0.000    0.000    0.000    0.000
   .fear12            0.000                               0.000    0.000    0.000    0.000
   .fear21     (c)    0.856    0.073   11.693    0.000    0.713    0.999    0.856    0.523
   .fear22     (c)    0.856    0.073   11.693    0.000    0.713    0.999    0.856    0.487
   .fear31     (d)    0.061    0.050    1.205    0.228   -0.038    0.160    0.061    0.036
   .fear32     (d)    0.061    0.050    1.205    0.228   -0.038    0.160    0.061    0.034
   .fear1    (g21)    4.790    0.107   44.737    0.000    4.580    5.000    3.304    3.304
   .fear2    (g22)    4.406    0.108   40.747    0.000    4.194    4.618    2.805    2.805

Variances:
                   Estimate  Std.Err  z-value  P(>|z|) ci.lower ci.upper   Std.lv  Std.all
   .fear11            0.651    0.090    7.206    0.000    0.474    0.828    0.651    0.236
   .fear21            0.828    0.080   10.347    0.000    0.671    0.985    0.828    0.309
   .fear31            1.516    0.092   16.544    0.000    1.337    1.696    1.516    0.537
   .fear12            0.595    0.088    6.739    0.000    0.422    0.769    0.595    0.194
   .fear22            0.910    0.087   10.507    0.000    0.740    1.080    0.910    0.295
   .fear32            1.642    0.110   14.969    0.000    1.427    1.857    1.642    0.517
   .fear1             2.081    0.150   13.915    0.000    1.788    2.374    0.990    0.990
   .fear2             2.448    0.145   16.873    0.000    2.164    2.732    0.992    0.992


Group 3 [3]:

Latent Variables:
                   Estimate  Std.Err  z-value  P(>|z|) ci.lower ci.upper   Std.lv  Std.all
  fear1 =~
    fear11            1.000                               1.000    1.000    1.783    0.833
    fear21     (a)    0.939    0.014   65.156    0.000    0.910    0.967    1.674    0.828
    fear31     (b)    0.788    0.012   66.529    0.000    0.765    0.811    1.405    0.822
  fear2 =~
    fear12            1.000                               1.000    1.000    1.691    0.824
    fear22     (a)    0.939    0.014   65.156    0.000    0.910    0.967    1.587    0.827
    fear32     (b)    0.788    0.012   66.529    0.000    0.765    0.811    1.332    0.803

Regressions:
                   Estimate  Std.Err  z-value  P(>|z|) ci.lower ci.upper   Std.lv  Std.all
```

```
  fear1 ~
    ageb       (j1)   -0.005    0.222   -0.023    0.982   -0.441    0.431   -0.003   -0.001
    male1      (j2)   -0.404    0.174   -2.322    0.020   -0.745   -0.063   -0.227   -0.113
    hedu       (j3)   -0.083    0.233   -0.355    0.722   -0.539    0.373   -0.046   -0.017
    lincome1   (j4)    0.389    0.191    2.038    0.042    0.015    0.762    0.218    0.107
    imm1       (j5)    0.320    0.232    1.382    0.167   -0.134    0.774    0.180    0.070
  fear2 ~
    ageb       (j1)   -0.005    0.222   -0.023    0.982   -0.441    0.431   -0.003   -0.001
    male1      (j2)   -0.404    0.174   -2.322    0.020   -0.745   -0.063   -0.239   -0.119
    hedu       (j3)   -0.083    0.233   -0.355    0.722   -0.539    0.373   -0.049   -0.018
    lincome1   (j4)    0.389    0.191    2.038    0.042    0.015    0.762    0.230    0.113
    imm1       (j5)    0.320    0.232    1.382    0.167   -0.134    0.774    0.189    0.074

Covariances:
                   Estimate  Std.Err  z-value  P(>|z|) ci.lower ci.upper   Std.lv  Std.all
 .fear11 ~~
   .fear12            0.275    0.149    1.848    0.065   -0.017    0.566    0.275    0.200
 .fear21 ~~
   .fear22            0.624    0.118    5.304    0.000    0.393    0.854    0.624    0.511
 .fear31 ~~
   .fear32            0.388    0.095    4.080    0.000    0.201    0.574    0.388    0.404
 .fear1 ~~
   .fear2             2.408    0.195   12.370    0.000    2.026    2.789    0.827    0.827

Intercepts:
                   Estimate  Std.Err  z-value  P(>|z|) ci.lower ci.upper   Std.lv  Std.all
   .fear11            0.000                               0.000    0.000    0.000    0.000
   .fear12            0.000                               0.000    0.000    0.000    0.000
   .fear21     (c)    0.856    0.073   11.693    0.000    0.713    0.999    0.856    0.424
   .fear22     (c)    0.856    0.073   11.693    0.000    0.713    0.999    0.856    0.446
   .fear31     (d)    0.061    0.050    1.205    0.228   -0.038    0.160    0.061    0.036
   .fear32     (d)    0.061    0.050    1.205    0.228   -0.038    0.160    0.061    0.037
   .fear1    (g31)    2.938    0.164   17.926    0.000    2.617    3.260    1.648    1.648
   .fear2    (g32)    2.612    0.161   16.225    0.000    2.297    2.928    1.545    1.545

Variances:
                   Estimate  Std.Err  z-value  P(>|z|) ci.lower ci.upper   Std.lv  Std.all
   .fear11            1.399    0.274    5.098    0.000    0.861    1.937    1.399    0.306
```

```
   .fear21            1.283    0.147    8.737    0.000    0.995    1.570    1.283    0.314
   .fear31            0.945    0.123    7.715    0.000    0.705    1.185    0.945    0.324
   .fear12            1.350    0.260    5.197    0.000    0.841    1.860    1.350    0.321
   .fear22            1.161    0.135    8.619    0.000    0.897    1.425    1.161    0.316
   .fear32            0.976    0.111    8.803    0.000    0.759    1.194    0.976    0.355
   .fear1             3.076    0.210   14.679    0.000    2.665    3.486    0.968    0.968
   .fear2             2.755    0.230   11.957    0.000    2.303    3.206    0.964    0.964


Group 4 [4]:

Latent Variables:
                   Estimate  Std.Err  z-value  P(>|z|) ci.lower ci.upper   Std.lv  Std.all
  fear1 =~
    fear11            1.000                               1.000    1.000    1.623    0.903
    fear21     (a)    0.939    0.014   65.156    0.000    0.910    0.967    1.523    0.843
    fear31     (b)    0.788    0.012   66.529    0.000    0.765    0.811    1.279    0.699
  fear2 =~
    fear12            1.000                               1.000    1.000    1.569    0.831
    fear22     (a)    0.939    0.014   65.156    0.000    0.910    0.967    1.473    0.812
    fear32     (b)    0.788    0.012   66.529    0.000    0.765    0.811    1.237    0.691

Regressions:
                   Estimate  Std.Err  z-value  P(>|z|) ci.lower ci.upper   Std.lv  Std.all
  fear1 ~
    ageb      (k1)    0.017    0.378    0.045    0.964   -0.724    0.759    0.011    0.003
    male1     (k2)   -0.463    0.223   -2.075    0.038   -0.901   -0.026   -0.286   -0.143
    hedu      (k3)    0.329    0.296    1.111    0.267   -0.251    0.908    0.203    0.084
    lincome1  (k4)   -0.122    0.232   -0.525    0.600   -0.576    0.333   -0.075   -0.037
    imm1      (k5)   -0.160    0.252   -0.634    0.526   -0.654    0.334   -0.098   -0.042
  fear2 ~
    ageb      (k1)    0.017    0.378    0.045    0.964   -0.724    0.759    0.011    0.003
    male1     (k2)   -0.463    0.223   -2.075    0.038   -0.901   -0.026   -0.295   -0.148
    hedu      (k3)    0.329    0.296    1.111    0.267   -0.251    0.908    0.209    0.087
    lincome1  (k4)   -0.122    0.232   -0.525    0.600   -0.576    0.333   -0.078   -0.038
    imm1      (k5)   -0.160    0.252   -0.634    0.526   -0.654    0.334   -0.102   -0.044

Covariances:
```

```
                   Estimate  Std.Err  z-value  P(>|z|) ci.lower ci.upper   Std.lv  Std.all
 .fear11 ~~
   .fear12            0.044    0.150    0.295    0.768   -0.250    0.339    0.044    0.055
 .fear21 ~~
   .fear22            0.338    0.118    2.870    0.004    0.107    0.570    0.338    0.330
 .fear31 ~~
   .fear32            0.768    0.156    4.926    0.000    0.462    1.073    0.768    0.454
 .fear1 ~~
   .fear2             1.850    0.241    7.682    0.000    1.378    2.322    0.753    0.753

Intercepts:
                   Estimate  Std.Err  z-value  P(>|z|) ci.lower ci.upper   Std.lv  Std.all
   .fear11            0.000                               0.000    0.000    0.000    0.000
   .fear12            0.000                               0.000    0.000    0.000    0.000
   .fear21      (c)   0.856    0.073   11.693    0.000    0.713    0.999    0.856    0.474
   .fear22      (c)   0.856    0.073   11.693    0.000    0.713    0.999    0.856    0.472
   .fear31      (d)   0.061    0.050    1.205    0.228   -0.038    0.160    0.061    0.033
   .fear32      (d)   0.061    0.050    1.205    0.228   -0.038    0.160    0.061    0.034
   .fear1     (g41)   4.875    0.204   23.941    0.000    4.476    5.274    3.004    3.004
   .fear2     (g42)   4.548    0.209   21.731    0.000    4.138    4.958    2.898    2.898

Variances:
                   Estimate  Std.Err  z-value  P(>|z|) ci.lower ci.upper   Std.lv  Std.all
   .fear11            0.597    0.174    3.435    0.001    0.256    0.938    0.597    0.185
   .fear21            0.942    0.152    6.192    0.000    0.644    1.240    0.942    0.289
   .fear31            1.709    0.169   10.086    0.000    1.377    2.041    1.709    0.511
   .fear12            1.105    0.300    3.687    0.000    0.518    1.693    1.105    0.310
   .fear22            1.119    0.167    6.715    0.000    0.793    1.446    1.119    0.340
   .fear32            1.676    0.219    7.657    0.000    1.247    2.105    1.676    0.523
   .fear1             2.543    0.271    9.372    0.000    2.011    3.074    0.966    0.966
   .fear2             2.373    0.261    9.093    0.000    1.861    2.884    0.963    0.963

Defined Parameters:
                   Estimate  Std.Err  z-value  P(>|z|) ci.lower ci.upper   Std.lv  Std.all
    did12            -0.156    0.056   -2.779    0.005   -0.265   -0.046   -0.147   -0.147
    did13            -0.213    0.070   -3.044    0.002   -0.351   -0.076   -0.543   -0.543
    did14            -0.213    0.101   -2.107    0.035   -0.411   -0.015   -0.540   -0.540
    did23            -0.058    0.081   -0.716    0.474   -0.216    0.100   -0.397   -0.397
```

```
    did24              -0.057    0.109   -0.528    0.598   -0.270    0.156   -0.394   -0.394
    did34               0.000    0.116    0.003    0.997   -0.228    0.229    0.003    0.003

>
> ################################################################################
>
> ##### 5 Additional model using infection status (tested positive) #####
> #####   at t1 and t2 as additional covariates (ptest1 and ptest2) #####
>
> sem2 <-'
+ group: [1]
+ fear1=~1*fear11+a*fear21+b*fear31
+ fear2=~1*fear12+a*fear22+b*fear32
+ fear11~0*1
+ fear12~0*1
+ fear21~c*1
+ fear22~c*1
+ fear31~d*1
+ fear32~d*1
+ fear1~g11*1
+ fear2~g12*1
+ fear11~~fear12
+ fear21~~fear22
+ fear31~~fear32
+ # control
+ fear1~h1*ageb+h2*male1+h3*hedu+h4*lincome1+h5*imm1+ptest1
+ fear2~h1*ageb+h2*male1+h3*hedu+h4*lincome1+h5*imm1+ptest2
+ group: [2]
+ fear1=~1*fear11+a*fear21+b*fear31
+ fear2=~1*fear12+a*fear22+b*fear32
+ fear11~0*1
+ fear12~0*1
+ fear21~c*1
+ fear22~c*1
+ fear31~d*1
+ fear32~d*1
+ fear1~g21*1
+ fear2~g22*1
```

```
+ fear11~~fear12
+ fear21~~fear22
+ fear31~~fear32
+ # control
+ fear1~i1*ageb+i2*male1+i3*hedu+i4*lincome1+i5*imm1+ptest1
+ fear2~i1*ageb+i2*male1+i3*hedu+i4*lincome1+i5*imm1+ptest2
+ group: [3]
+ fear1=~1*fear11+a*fear21+b*fear31
+ fear2=~1*fear12+a*fear22+b*fear32
+ fear11~0*1
+ fear12~0*1
+ fear21~c*1
+ fear22~c*1
+ fear31~d*1
+ fear32~d*1
+ fear1~g31*1
+ fear2~g32*1
+ fear11~~fear12
+ fear21~~fear22
+ fear31~~fear32
+ # control
+ fear1~j1*ageb+j2*male1+j3*hedu+j4*lincome1+j5*imm1+ptest1
+ fear2~j1*ageb+j2*male1+j3*hedu+j4*lincome1+j5*imm1+ptest2
+ group: [4]
+ fear1=~1*fear11+a*fear21+b*fear31
+ fear2=~1*fear12+a*fear22+b*fear32
+ fear11~0*1
+ fear12~0*1
+ fear21~c*1
+ fear22~c*1
+ fear31~d*1
+ fear32~d*1
+ fear1~g41*1
+ fear2~g42*1
+ fear11~~fear12
+ fear21~~fear22
+ fear31~~fear32
+ # control
```

```
+ fear1~k1*ageb+k2*male1+k3*hedu+k4*lincome1+k5*imm1+ptest1
+ fear2~k1*ageb+k2*male1+k3*hedu+k4*lincome1+k5*imm1+ptest2
+ ### latent mean differences-in-differences
+ # group 1 vs group 2
+ did12 := (g12-g11)-(g22-g21)
+ # group 1 vs group 3
+ did13 := (g12-g11)-(g32-g31)
+ # group 1 vs group 4
+ did14 := (g12-g11)-(g42-g41)
+ # group 2 vs group 3
+ did23 := (g22-g21)-(g32-g31)
+ # group 2 vs group 4
+ did24 := (g22-g21)-(g42-g41)
+ # group 3 vs group 4
+ did34 := (g32-g31)-(g42-g41)
+ '
> fsem2<-sem(sem2,data=fear_data,missing="FIML",estimator="MLR",group="groups")
Warnmeldungen:
1: In lav_data_full(data = data, group = group, cluster = cluster,  :
  lavaan WARNING: 75 cases were deleted in group 1 due to missing values in
            exogenous variable(s), while fixed.x = TRUE.
2: In lav_data_full(data = data, group = group, cluster = cluster,  :
  lavaan WARNING: 18 cases were deleted in group 2 due to missing values in
            exogenous variable(s), while fixed.x = TRUE.
3: In lav_data_full(data = data, group = group, cluster = cluster,  :
  lavaan WARNING: 27 cases were deleted in group 3 due to missing values in
            exogenous variable(s), while fixed.x = TRUE.
4: In lav_data_full(data = data, group = group, cluster = cluster,  :
  lavaan WARNING: 20 cases were deleted in group 4 due to missing values in
            exogenous variable(s), while fixed.x = TRUE.
> summary(fsem2,standardized=TRUE,fit.measures=TRUE,ci=TRUE)
lavaan 0.6-9 ended normally after 257 iterations

  Estimator                                         ML
  Optimization method                           NLMINB
  Number of model parameters                       136
  Number of equality constraints                    48
```

```
  Number of observations per group:               Used       Total
    1                                             2064        2139
    2                                              665         683
    3                                              372         399
    4                                              187         207
  Number of missing patterns per group:
    1                                                8
    2                                                2
    3                                                9
    4                                                4

Model Test User Model:
                                              Standard      Robust
  Test Statistic                               507.308     487.785
  Degrees of freedom                               188         188
  P-value (Chi-square)                           0.000       0.000
  Scaling correction factor                                  1.040
       Yuan-Bentler correction (Mplus variant)
  Test statistic for each group:
    1                                          179.346     172.444
    2                                          111.222     106.942
    3                                          140.928     135.504
    4                                           75.813      72.895

Model Test Baseline Model:

  Test statistic                             12687.691   10633.462
  Degrees of freedom                               228         228
  P-value                                        0.000       0.000
  Scaling correction factor                                  1.193

User Model versus Baseline Model:

  Comparative Fit Index (CFI)                    0.974       0.971
  Tucker-Lewis Index (TLI)                       0.969       0.965

  Robust Comparative Fit Index (CFI)                         0.975
  Robust Tucker-Lewis Index (TLI)                            0.970
```

```
Loglikelihood and Information Criteria:

  Loglikelihood user model (H0)             -33201.099  -33201.099
  Scaling correction factor                                  0.862
      for the MLR correction
  Loglikelihood unrestricted model (H1)     -32947.445  -32947.445
  Scaling correction factor                                  1.133
      for the MLR correction

  Akaike (AIC)                               66578.198   66578.198
  Bayesian (BIC)                             67114.826   67114.826
  Sample-size adjusted Bayesian (BIC)        66835.210   66835.210

Root Mean Square Error of Approximation:

  RMSEA                                          0.045       0.044
  90 Percent confidence interval - lower         0.041       0.039
  90 Percent confidence interval - upper         0.050       0.049
  P-value RMSEA <= 0.05                          0.941       0.981

  Robust RMSEA                                               0.045
  90 Percent confidence interval - lower                     0.040
  90 Percent confidence interval - upper                     0.050

Standardized Root Mean Square Residual:

  SRMR                                           0.031       0.031

Parameter Estimates:

  Standard errors                             Sandwich
  Information bread                           Observed
  Observed information based on                Hessian


Group 1 [1]:
```

```
Latent Variables:
                   Estimate  Std.Err  z-value  P(>|z|) ci.lower ci.upper   Std.lv  Std.all
  fear1 =~
    fear11            1.000                               1.000    1.000    1.502    0.860
    fear21     (a)    0.936    0.015   63.632    0.000    0.907    0.965    1.406    0.826
    fear31     (b)    0.788    0.012   64.095    0.000    0.764    0.813    1.184    0.666
  fear2 =~
    fear12            1.000                               1.000    1.000    1.678    0.909
    fear22     (a)    0.936    0.015   63.632    0.000    0.907    0.965    1.571    0.843
    fear32     (b)    0.788    0.012   64.095    0.000    0.764    0.813    1.323    0.748

Regressions:
                   Estimate  Std.Err  z-value  P(>|z|) ci.lower ci.upper   Std.lv  Std.all
  fear1 ~
    ageb      (h1)    0.230    0.080    2.888    0.004    0.074    0.386    0.153    0.067
    male1     (h2)   -0.246    0.070   -3.523    0.000   -0.383   -0.109   -0.164   -0.081
    hedu      (h3)    0.120    0.081    1.481    0.139   -0.039    0.279    0.080    0.034
    lincome1  (h4)    0.041    0.082    0.498    0.618   -0.120    0.202    0.027    0.012
    imm1      (h5)    0.150    0.089    1.688    0.091   -0.024    0.324    0.100    0.037
    ptest1            0.168    0.135    1.245    0.213   -0.096    0.432    0.112    0.021
  fear2 ~
    ageb      (h1)    0.230    0.080    2.888    0.004    0.074    0.386    0.137    0.060
    male1     (h2)   -0.246    0.070   -3.523    0.000   -0.383   -0.109   -0.147   -0.073
    hedu      (h3)    0.120    0.081    1.481    0.139   -0.039    0.279    0.072    0.031
    lincome1  (h4)    0.041    0.082    0.498    0.618   -0.120    0.202    0.024    0.011
    imm1      (h5)    0.150    0.089    1.688    0.091   -0.024    0.324    0.089    0.033
    ptest2            0.309    0.143    2.164    0.030    0.029    0.588    0.184    0.039

Covariances:
                   Estimate  Std.Err  z-value  P(>|z|) ci.lower ci.upper   Std.lv  Std.all
 .fear11 ~~
   .fear12           -0.033    0.042   -0.789    0.430   -0.115    0.049   -0.033   -0.048
 .fear21 ~~
   .fear22            0.424    0.040   10.622    0.000    0.346    0.502    0.424    0.440
 .fear31 ~~
   .fear32            0.626    0.046   13.600    0.000    0.536    0.716    0.626    0.402
 .fear1 ~~
   .fear2             1.972    0.077   25.734    0.000    1.822    2.122    0.791    0.791
```

```
Intercepts:
                   Estimate  Std.Err  z-value  P(>|z|) ci.lower ci.upper   Std.lv  Std.all
   .fear11            0.000                               0.000    0.000    0.000    0.000
   .fear12            0.000                               0.000    0.000    0.000    0.000
   .fear21     (c)    0.879    0.075   11.719    0.000    0.732    1.026    0.879    0.516
   .fear22     (c)    0.879    0.075   11.719    0.000    0.732    1.026    0.879    0.471
   .fear31     (d)    0.050    0.053    0.954    0.340   -0.053    0.154    0.050    0.028
   .fear32     (d)    0.050    0.053    0.954    0.340   -0.053    0.154    0.050    0.028
   .fear1    (g11)    4.834    0.067   71.808    0.000    4.702    4.966    3.217    3.217
   .fear2    (g12)    4.284    0.069   61.842    0.000    4.148    4.420    2.553    2.553

Variances:
                   Estimate  Std.Err  z-value  P(>|z|) ci.lower ci.upper   Std.lv  Std.all
   .fear11            0.794    0.070   11.378    0.000    0.658    0.931    0.794    0.260
   .fear21            0.920    0.049   18.655    0.000    0.823    1.016    0.920    0.317
   .fear31            1.757    0.062   28.539    0.000    1.636    1.878    1.757    0.556
   .fear12            0.590    0.059    9.987    0.000    0.474    0.706    0.590    0.173
   .fear22            1.008    0.054   18.680    0.000    0.902    1.113    1.008    0.290
   .fear32            1.377    0.058   23.875    0.000    1.264    1.490    1.377    0.440
   .fear1             2.229    0.087   25.481    0.000    2.057    2.400    0.987    0.987
   .fear2             2.785    0.091   30.771    0.000    2.608    2.963    0.989    0.989


Group 2 [2]:

Latent Variables:
                   Estimate  Std.Err  z-value  P(>|z|) ci.lower ci.upper   Std.lv  Std.all
  fear1 =~
    fear11            1.000                               1.000    1.000    1.447    0.876
    fear21     (a)    0.936    0.015   63.632    0.000    0.907    0.965    1.354    0.831
    fear31     (b)    0.788    0.012   64.095    0.000    0.764    0.813    1.140    0.678
  fear2 =~
    fear12            1.000                               1.000    1.000    1.567    0.900
    fear22     (a)    0.936    0.015   63.632    0.000    0.907    0.965    1.467    0.836
    fear32     (b)    0.788    0.012   64.095    0.000    0.764    0.813    1.236    0.696

Regressions:
```

```
                   Estimate  Std.Err  z-value  P(>|z|) ci.lower ci.upper   Std.lv  Std.all
  fear1 ~
    ageb      (i1)    0.171    0.121    1.411    0.158   -0.066    0.408    0.118    0.058
    male1     (i2)   -0.083    0.119   -0.704    0.482   -0.316    0.149   -0.058   -0.029
    hedu      (i3)    0.021    0.145    0.148    0.883   -0.262    0.305    0.015    0.006
    lincome1  (i4)    0.236    0.137    1.723    0.085   -0.032    0.504    0.163    0.070
    imm1      (i5)   -0.050    0.148   -0.338    0.735   -0.339    0.240   -0.034   -0.012
    ptest1            0.341    0.207    1.642    0.101   -0.066    0.747    0.235    0.046
  fear2 ~
    ageb      (i1)    0.171    0.121    1.411    0.158   -0.066    0.408    0.109    0.054
    male1     (i2)   -0.083    0.119   -0.704    0.482   -0.316    0.149   -0.053   -0.026
    hedu      (i3)    0.021    0.145    0.148    0.883   -0.262    0.305    0.014    0.006
    lincome1  (i4)    0.236    0.137    1.723    0.085   -0.032    0.504    0.150    0.065
    imm1      (i5)   -0.050    0.148   -0.338    0.735   -0.339    0.240   -0.032   -0.011
    ptest2           -0.076    0.224   -0.341    0.733   -0.515    0.362   -0.049   -0.011

Covariances:
                   Estimate  Std.Err  z-value  P(>|z|) ci.lower ci.upper   Std.lv  Std.all
 .fear11 ~~
   .fear12            0.036    0.063    0.565    0.572   -0.088    0.160    0.036    0.059
 .fear21 ~~
   .fear22            0.340    0.067    5.093    0.000    0.209    0.471    0.340    0.390
 .fear31 ~~
   .fear32            0.665    0.078    8.500    0.000    0.512    0.818    0.665    0.422
 .fear1 ~~
   .fear2             1.742    0.131   13.281    0.000    1.485    2.000    0.776    0.776

Intercepts:
                   Estimate  Std.Err  z-value  P(>|z|) ci.lower ci.upper   Std.lv  Std.all
   .fear11            0.000                               0.000    0.000    0.000    0.000
   .fear12            0.000                               0.000    0.000    0.000    0.000
   .fear21     (c)    0.879    0.075   11.719    0.000    0.732    1.026    0.879    0.539
   .fear22     (c)    0.879    0.075   11.719    0.000    0.732    1.026    0.879    0.501
   .fear31     (d)    0.050    0.053    0.954    0.340   -0.053    0.154    0.050    0.030
   .fear32     (d)    0.050    0.053    0.954    0.340   -0.053    0.154    0.050    0.028
   .fear1    (g21)    4.803    0.107   44.723    0.000    4.592    5.013    3.320    3.320
   .fear2    (g22)    4.425    0.108   40.858    0.000    4.213    4.638    2.823    2.823
```

```
Variances:
                   Estimate  Std.Err  z-value  P(>|z|) ci.lower ci.upper   Std.lv  Std.all
   .fear11            0.637    0.087    7.292    0.000    0.466    0.808    0.637    0.233
   .fear21            0.823    0.081   10.143    0.000    0.664    0.982    0.823    0.310
   .fear31            1.528    0.093   16.430    0.000    1.346    1.710    1.528    0.540
   .fear12            0.575    0.089    6.470    0.000    0.401    0.750    0.575    0.190
   .fear22            0.924    0.088   10.495    0.000    0.752    1.097    0.924    0.300
   .fear32            1.627    0.110   14.739    0.000    1.410    1.843    1.627    0.516
   .fear1             2.070    0.150   13.817    0.000    1.777    2.364    0.989    0.989
   .fear2             2.436    0.146   16.636    0.000    2.149    2.723    0.992    0.992


Group 3 [3]:

Latent Variables:
                   Estimate  Std.Err  z-value  P(>|z|) ci.lower ci.upper   Std.lv  Std.all
  fear1 =~
    fear11            1.000                               1.000    1.000    1.790    0.843
    fear21     (a)    0.936    0.015   63.632    0.000    0.907    0.965    1.675    0.827
    fear31     (b)    0.788    0.012   64.095    0.000    0.764    0.813    1.411    0.817
  fear2 =~
    fear12            1.000                               1.000    1.000    1.707    0.829
    fear22     (a)    0.936    0.015   63.632    0.000    0.907    0.965    1.598    0.824
    fear32     (b)    0.788    0.012   64.095    0.000    0.764    0.813    1.346    0.799

Regressions:
                   Estimate  Std.Err  z-value  P(>|z|) ci.lower ci.upper   Std.lv  Std.all
  fear1 ~
    ageb      (j1)    0.032    0.231    0.139    0.890   -0.421    0.485    0.018    0.006
    male1     (j2)   -0.443    0.181   -2.448    0.014   -0.798   -0.088   -0.248   -0.123
    hedu      (j3)   -0.072    0.248   -0.292    0.770   -0.559    0.414   -0.040   -0.015
    lincome1  (j4)    0.395    0.200    1.976    0.048    0.003    0.787    0.221    0.108
    imm1      (j5)    0.259    0.234    1.106    0.269   -0.200    0.718    0.145    0.057
    ptest1            0.291    0.391    0.744    0.457   -0.475    1.057    0.162    0.031
  fear2 ~
    ageb      (j1)    0.032    0.231    0.139    0.890   -0.421    0.485    0.019    0.007
    male1     (j2)   -0.443    0.181   -2.448    0.014   -0.798   -0.088   -0.260   -0.129
    hedu      (j3)   -0.072    0.248   -0.292    0.770   -0.559    0.414   -0.042   -0.015
```

```
    lincome1   (j4)    0.395    0.200    1.976    0.048    0.003    0.787    0.232    0.113
    imm1       (j5)    0.259    0.234    1.106    0.269   -0.200    0.718    0.152    0.060
    ptest2            -0.269    0.367   -0.734    0.463   -0.989    0.450   -0.158   -0.034

Covariances:
                   Estimate  Std.Err  z-value  P(>|z|) ci.lower ci.upper   Std.lv  Std.all
 .fear11 ~~
   .fear12             0.246    0.148    1.666    0.096   -0.043    0.535    0.246    0.187
 .fear21 ~~
   .fear22             0.640    0.122    5.229    0.000    0.400    0.880    0.640    0.512
 .fear31 ~~
   .fear32             0.409    0.101    4.049    0.000    0.211    0.607    0.409    0.405
 .fear1 ~~
   .fear2              2.441    0.203   12.030    0.000    2.044    2.839    0.829    0.829

Intercepts:
                   Estimate  Std.Err  z-value  P(>|z|) ci.lower ci.upper   Std.lv  Std.all
   .fear11             0.000                               0.000    0.000    0.000    0.000
   .fear12             0.000                               0.000    0.000    0.000    0.000
   .fear21      (c)    0.879    0.075   11.719    0.000    0.732    1.026    0.879    0.434
   .fear22      (c)    0.879    0.075   11.719    0.000    0.732    1.026    0.879    0.453
   .fear31      (d)    0.050    0.053    0.954    0.340   -0.053    0.154    0.050    0.029
   .fear32      (d)    0.050    0.053    0.954    0.340   -0.053    0.154    0.050    0.030
   .fear1     (g31)    3.021    0.173   17.512    0.000    2.683    3.359    1.688    1.688
   .fear2     (g32)    2.701    0.170   15.846    0.000    2.367    3.036    1.582    1.582

Variances:
                   Estimate  Std.Err  z-value  P(>|z|) ci.lower ci.upper   Std.lv  Std.all
   .fear11             1.308    0.270    4.847    0.000    0.779    1.837    1.308    0.290
   .fear21             1.296    0.151    8.556    0.000    0.999    1.593    1.296    0.316
   .fear31             0.995    0.130    7.648    0.000    0.740    1.249    0.995    0.333
   .fear12             1.325    0.257    5.150    0.000    0.821    1.830    1.325    0.313
   .fear22             1.206    0.142    8.515    0.000    0.928    1.483    1.206    0.321
   .fear32             1.024    0.118    8.680    0.000    0.793    1.255    1.024    0.361
   .fear1              3.095    0.217   14.280    0.000    2.671    3.520    0.966    0.966
   .fear2              2.802    0.240   11.673    0.000    2.332    3.273    0.962    0.962
```

```
Group 4 [4]:

Latent Variables:
                   Estimate  Std.Err  z-value  P(>|z|) ci.lower ci.upper   Std.lv  Std.all
  fear1 =~
    fear11            1.000                               1.000    1.000    1.624    0.896
    fear21     (a)    0.936    0.015   63.632    0.000    0.907    0.965    1.520    0.842
    fear31     (b)    0.788    0.012   64.095    0.000    0.764    0.813    1.280    0.690
  fear2 =~
    fear12            1.000                               1.000    1.000    1.601    0.848
    fear22     (a)    0.936    0.015   63.632    0.000    0.907    0.965    1.499    0.812
    fear32     (b)    0.788    0.012   64.095    0.000    0.764    0.813    1.262    0.710

Regressions:
                   Estimate  Std.Err  z-value  P(>|z|) ci.lower ci.upper   Std.lv  Std.all
  fear1 ~
    ageb      (k1)   -0.012    0.409   -0.030    0.976   -0.815    0.790   -0.008   -0.002
    male1     (k2)   -0.485    0.235   -2.066    0.039   -0.945   -0.025   -0.299   -0.149
    hedu      (k3)    0.411    0.304    1.353    0.176   -0.184    1.007    0.253    0.106
    lincome1  (k4)   -0.096    0.246   -0.390    0.696   -0.577    0.386   -0.059   -0.029
    imm1      (k5)   -0.145    0.269   -0.537    0.592   -0.673    0.384   -0.089   -0.038
    ptest1            0.346    0.371    0.933    0.351   -0.382    1.075    0.213    0.064
  fear2 ~
    ageb      (k1)   -0.012    0.409   -0.030    0.976   -0.815    0.790   -0.008   -0.002
    male1     (k2)   -0.485    0.235   -2.066    0.039   -0.945   -0.025   -0.303   -0.151
    hedu      (k3)    0.411    0.304    1.353    0.176   -0.184    1.007    0.257    0.107
    lincome1  (k4)   -0.096    0.246   -0.390    0.696   -0.577    0.386   -0.060   -0.029
    imm1      (k5)   -0.145    0.269   -0.537    0.592   -0.673    0.384   -0.090   -0.039
    ptest2           -0.125    0.364   -0.344    0.731   -0.839    0.588   -0.078   -0.025

Covariances:
                   Estimate  Std.Err  z-value  P(>|z|) ci.lower ci.upper   Std.lv  Std.all
 .fear11 ~~
   .fear12            0.125    0.153    0.816    0.414   -0.175    0.424    0.125    0.155
 .fear21 ~~
   .fear22            0.391    0.127    3.079    0.002    0.142    0.639    0.391    0.372
 .fear31 ~~
   .fear32            0.798    0.164    4.865    0.000    0.477    1.120    0.798    0.475
```

```
 .fear1 ~~
   .fear2              1.836    0.247    7.444    0.000    1.352    2.319    0.739    0.739

Intercepts:
                   Estimate  Std.Err  z-value  P(>|z|) ci.lower ci.upper   Std.lv  Std.all
   .fear11            0.000                               0.000    0.000    0.000    0.000
   .fear12            0.000                               0.000    0.000    0.000    0.000
   .fear21      (c)   0.879    0.075   11.719    0.000    0.732    1.026    0.879    0.487
   .fear22      (c)   0.879    0.075   11.719    0.000    0.732    1.026    0.879    0.476
   .fear31      (d)   0.050    0.053    0.954    0.340   -0.053    0.154    0.050    0.027
   .fear32      (d)   0.050    0.053    0.954    0.340   -0.053    0.154    0.050    0.028
   .fear1     (g41)   4.858    0.213   22.827    0.000    4.441    5.275    2.992    2.992
   .fear2     (g42)   4.570    0.221   20.727    0.000    4.138    5.003    2.854    2.854

Variances:
                   Estimate  Std.Err  z-value  P(>|z|) ci.lower ci.upper   Std.lv  Std.all
   .fear11            0.647    0.184    3.518    0.000    0.287    1.008    0.647    0.197
   .fear21            0.950    0.165    5.753    0.000    0.626    1.273    0.950    0.291
   .fear31            1.802    0.183    9.855    0.000    1.443    2.160    1.802    0.524
   .fear12            1.001    0.290    3.450    0.001    0.432    1.570    1.001    0.281
   .fear22            1.163    0.179    6.501    0.000    0.812    1.513    1.163    0.341
   .fear32            1.569    0.226    6.950    0.000    1.127    2.011    1.569    0.496
   .fear1             2.515    0.277    9.074    0.000    1.971    3.058    0.954    0.954
   .fear2             2.454    0.271    9.055    0.000    1.923    2.985    0.957    0.957

Defined Parameters:
                   Estimate  Std.Err  z-value  P(>|z|) ci.lower ci.upper   Std.lv  Std.all
    did12            -0.172    0.057   -2.997    0.003   -0.285   -0.060   -0.168   -0.168
    did13            -0.230    0.073   -3.128    0.002   -0.374   -0.086   -0.559   -0.559
    did14            -0.262    0.113   -2.319    0.020   -0.483   -0.041   -0.527   -0.527
    did23            -0.057    0.084   -0.686    0.493   -0.222    0.107   -0.391   -0.391
    did24            -0.089    0.120   -0.745    0.456   -0.324    0.146   -0.359   -0.359
    did34            -0.032    0.128   -0.249    0.803   -0.283    0.219    0.033    0.033

>
> ################################################################################
> ################################################################################
```
